# Trace metals in Northern New England streams: Evaluating the role of road salt across broad spatial scales with synoptic snapshots

**DOI:** 10.1371/journal.pone.0212011

**Published:** 2019-02-13

**Authors:** Jessica F. Wilhelm, Daniel J. Bain, Mark B. Green, Kathleen F. Bush, William H. McDowell

**Affiliations:** 1 Center for the Environment, Plymouth State University, Plymouth, New Hampshire, United States of America; 2 Department of Geology and Environmental Science, University of Pittsburgh, Pittsburgh, Pennsylvania, United States of America; 3 Northern Research Station, United States Forest Service, Durham, New Hampshire, United States of America; 4 New Hampshire Department of Health and Human Services, Concord, New Hampshire, United States of America; 5 Natural Resources and the Environment, University of New Hampshire, Durham, New Hampshire, United States of America; Zhongnan University of Economics and Law, CHINA

## Abstract

Mobilization of trace metals from soils to surface waters can impact both human and ecosystem health. This study resamples a water sample archive to explore the spatial pattern of streamwater total concentrations of arsenic, cadmium, copper, lead, and zinc and their associations with biogeochemical controls in northern New England. Road deicing appears to result in elevated trace metal concentrations, as trace metal concentrations are strongly related to sodium concentrations and are most elevated when the sodium: chloride ratio is near 1.0 (~halite). Our results are consistent with previous laboratory and field studies that indicate cation exchange as a metal mobilization mechanism when road salt is applied to soils containing metals. This study also documents associations among sodium, chloride, dissolved organic carbon, iron, and metal concentrations, suggesting cation exchange mechanisms related to road deicing are not the only mechanisms that increase trace metal concentrations in surface waters. In addition to cation exchange, this study considers dissolved organic carbon complexation and oxidation-reduction conditions affecting metal mobility from soils in a salt-rich environment. These observations demonstrate that road deicing has the potential to increase streamwater trace metal concentrations across broad spatial scales and increase risks to human and ecosystem health.

## Introduction

Salinization of surface waters occurs across seasonally snow-covered areas of the world through the practice of road-deicing [1], and has important consequences for water quality [2–4] and aquatic ecosystems [1,5,6]. Chemical road deicers accumulate in groundwater [7,8], increasing the total concentration of major cations in groundwater throughout the year [9]. The higher concentrations of chloride (Cl) and sodium (Na) cause physiological stresses to aquatic organisms, and thus degrade aquatic ecosystems [4]. Salinization due to deicers also impairs drinking water [10], for example, some groundwater aquifers contain Na concentrations that are unsafe for consumers with high blood pressure [11]. Further, recent work suggests that elevated Cl in water increases corrosivity of drinking water infrastructure and may contribute to elevated trace metals in drinking water (e.g. lead, copper) [12].

**Fig 1:**
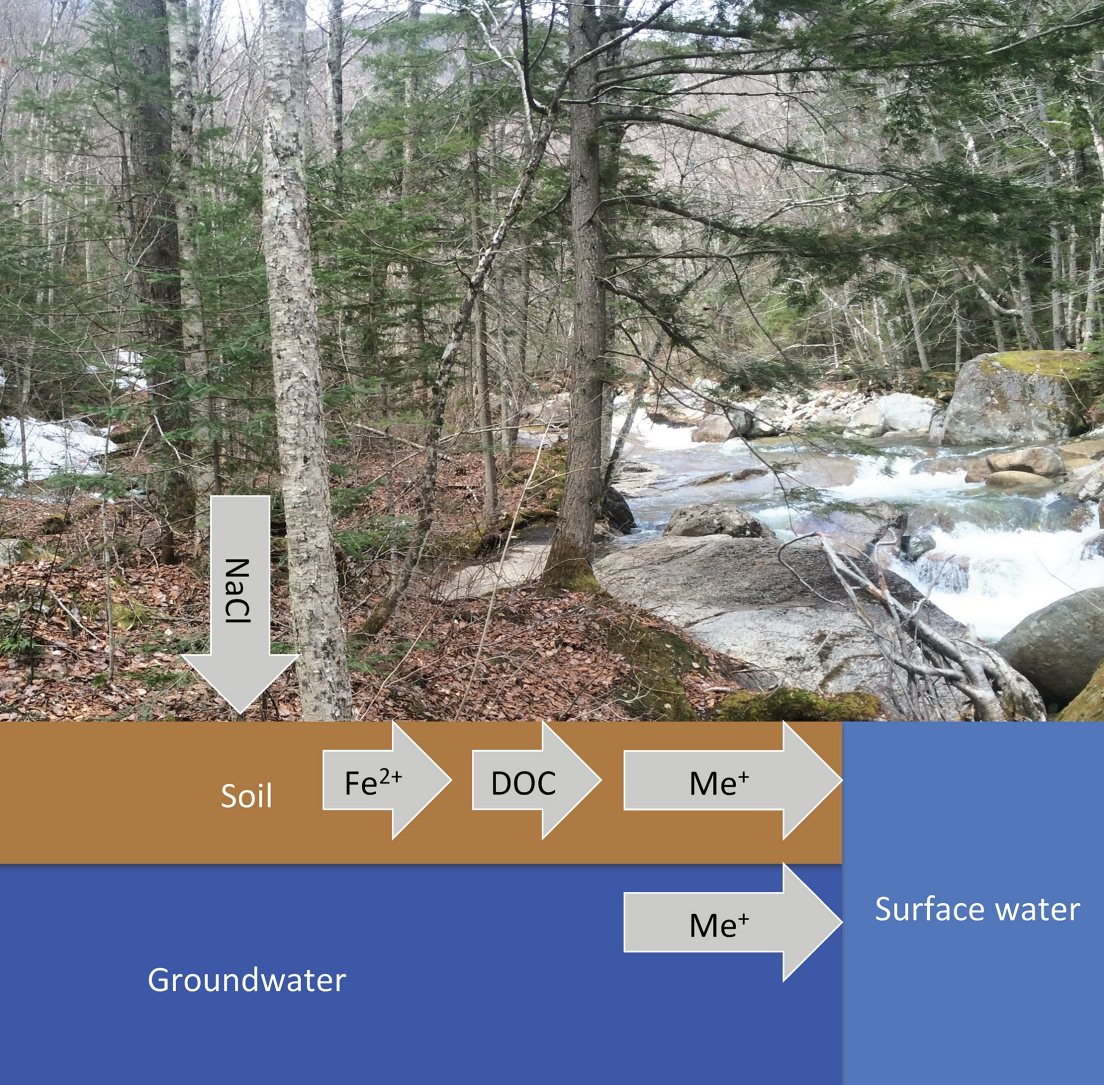
Associations among sodium, chloride, dissolved organic carbon, iron, and metal concentrations. This study considers cation exchange, dissolved organic carbon complexation, and oxidation-reduction conditions affecting metal mobility from soils in a salt-rich environment.

Deicer-driven salinization also impacts soils. Many studies have demonstrated the impact of salinization on soil cation exchange dynamics [13,14], which can mobilize trace metals from soils [15,16]. Soils with sorbed trace metals–either natural concentrations or concentrations enhanced due to human activities–are susceptible to altered ion exchange processes and mobilization [17]. Metal mobility is also enhanced by interactions among deicers, dissolved organic matter, and colloidal materials [15,17] (Fig 1).

Soils serve as an important regulator of trace metal dynamics, storing a range of potentially toxic metals. High concentrations of cations, particularly Na introduced during deicing, can displace metals via cation exchange and interact with dissolved organic carbon (DOC) and colloids to make soil metals available for potential transport to waters further downstream. In particular, Amrhein et al. [15] assessed trace metal mobility in roadside soils from cold weather climates using soil columns leached with road salt chemicals. They found metal concentrations increased during flushing by fresh water following exposure to NaCl. This simulated snowmelt seemed to mobilize low molecular weight DOC and bound metals. The fresh water flush contained loads of trace metals that were greater than those in the experimental high salt flush.

**Fig 2:**
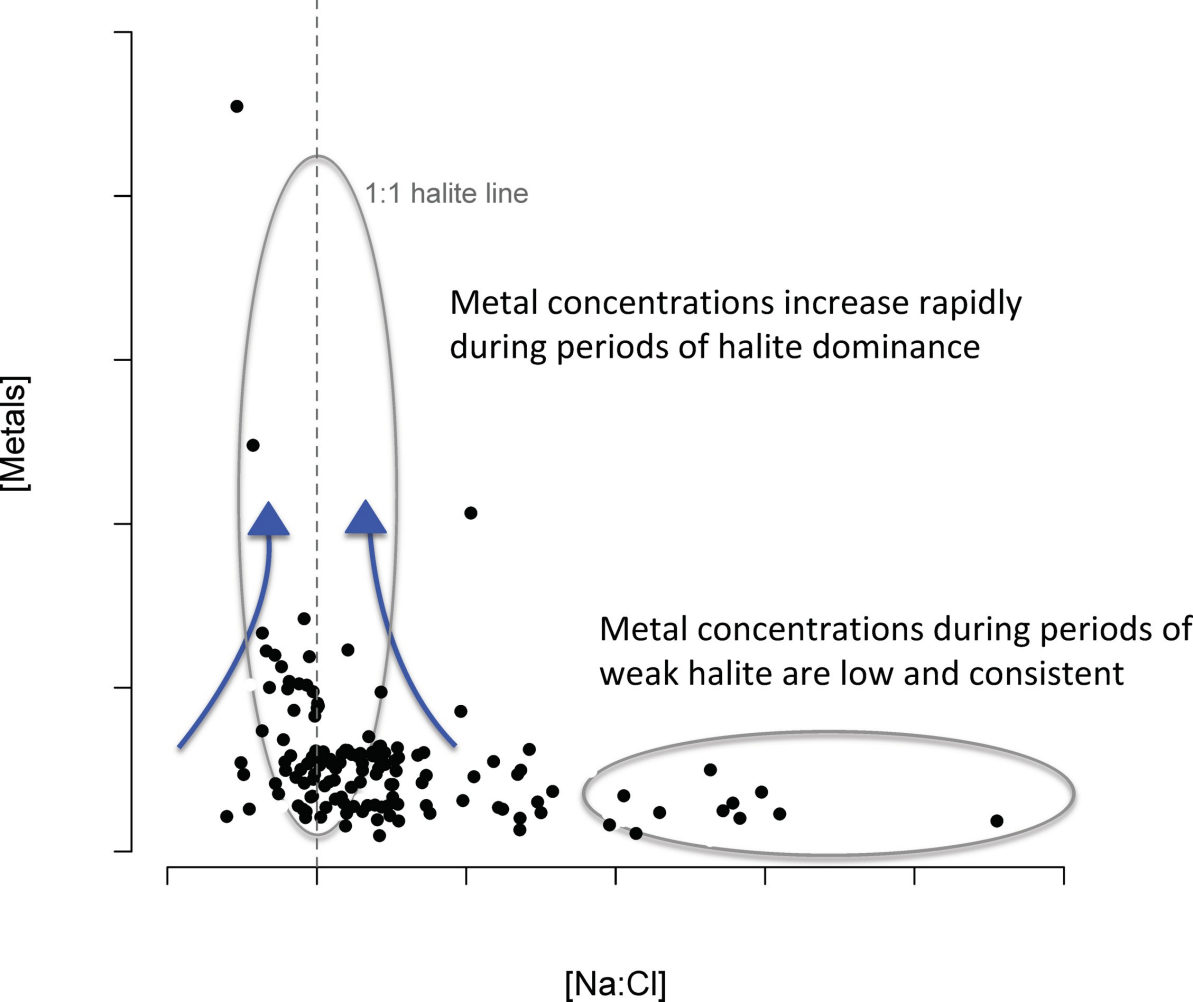
Metal concentrations during periods of fluctuating halite levels. The sites representing the tail denote metal concentrations when higher Na^+^ is released from exchange sites, relative to lower Cl^-^ flushed following road salt inputs. Corresponding Cl^-^ is quickly flushed through the system following input, and therefore absent in periods not dominated by halite. The sites near the 1:1 halite peak, i.e. equal Na^+^ and Cl^-^, suggest high dissolved metal flux associated with periods of high halite inputs.

The vast majority of deicer salinization and soil studies have been conducted either in the laboratory [15,18] or at limited spatial scales [17,19–21], and the impact of these salinization processes on regional streamwater quality remains poorly understood. This study documents total trace metal concentrations across a large spatial scale in Northern New England, using an archive of synoptically sampled surface waters, and compares the spatial variability of trace metal concentrations with indicators of chemical deicers (Fig 2). Given the archival nature of the samples, the impacts of storage conditions are assessed as part of this analysis. Strong co-variance between the amount of urbanization and chemical indicators of road salt has been documented in this region previously [9,22]. Therefore this study focuses on soil-streamwater processes in areas impacted by road salt.

## Methods

In 2013 approximately 70 sites in northern New England were sampled synoptically in May, June, and July (sampling and storage methods detailed below). All sampling sites are located at road crossings of flowing waters. By New Hampshire State law, all navigable waters (broadly defined) are public spaces (*Concord Mfg*. *Co*. *v*. *Robertson*, 25 A. 718, 720 (N.H. 1890)) and these public spaces are accessible via road right of ways (*Hartford v*. *Town of Gilmanton*, 101 N.H. 424 (N.H. 1959)). Therefore, special collection permissions were not required for any of the locations. Further, this study did not involve endangered or protected species. We evaluated concentrations of As, Cd, Cu, Pb, and Zn in the archived samples and compared these concentrations with road salt indicators (Na, Cl, and Na: Cl), water quality parameters (DOC), and redox indicators, iron (Fe).

### Site distribution

**Fig 3:**
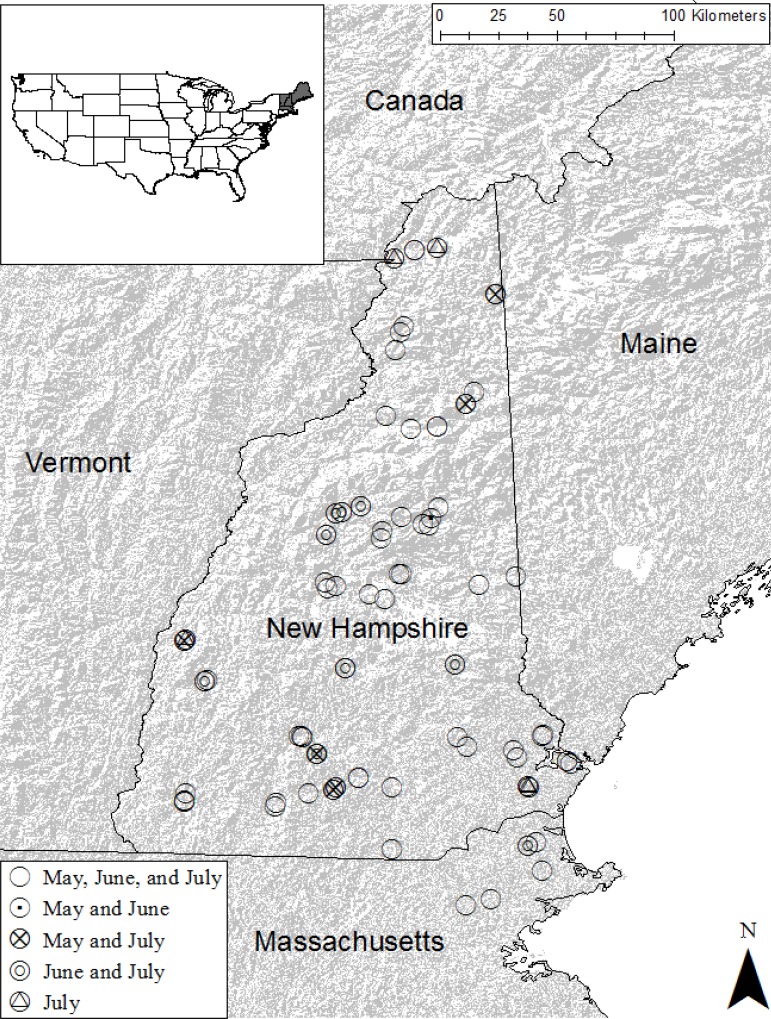
Site map. Sites in Massachusetts and New Hampshire evaluated for metal concentrations and biogeochemistry.

Sites were located in New Hampshire and northern Massachusetts and associated with the citizen science Lotic Volunteer Temperature, Electrical Conductivity, and Stage (LoVoTECS) sensing network [23] (Fig 3). Sites spanned a range of conditions including basin size, elevation, and land cover. Catchments ranged from 1.5 km^2^ to 2646 km^2^. Sample collection sites included four major New England rivers and their tributaries: the upper and lower Androscoggin, the Saco, the Merrimack, and the upper Connecticut. Study area climate is temperate; average July highs ranging between 24°C and 28°C, and average January lows ranging between -20°C and -9°C. Mean annual precipitation ranges between 40 cm and 118 cm [24]. The regional elevation ranges from 0 (MSL) to the highest point, Mount Washington, at 1,917 meters.

The bedrock geology of the greater New Hampshire region can be broadly separated into three composite terranes: the Grenville, Central Maine, and Nashoba-Casco-Miramichi [25]. Metasedimentary materials, found particularly in the southwestern part of the state, dominate most of New Hampshire. To the west, calc-silicate metasediments, and volcanic rocks and granitic gneisses are also important bed rock types [25].

### Water sampling

Three synoptic sampling events were performed May 14th (n = 61), June 11th (n = 66), and July 16th (n = 77) 2013. Although most sites were sampled on all three-sample collection dates, not all sites were sampled on all three dates. This limits our ability to interpret temporal patterns. All samples were collected within a 12-hour period on the sampling date. Grab samples were collected 1 to 2 meters from the stream or riverbank. One-liter polypropylene sample vials were rinsed three times with filtered stream water and 30 mL borosilicate glass vials were rinsed three times with stream water before sample collection. One-liter polypropylene samples were field filtered and used for major ion and DOC analyses. The samples collected in 30 mL borosilicate glass vials were intended for water isotope analysis and therefore were neither filtered nor acidified at the time of collection. Immediately following collection, the samples were placed on ice, and stored at 4°C upon return to the laboratory.

**Fig 4:**
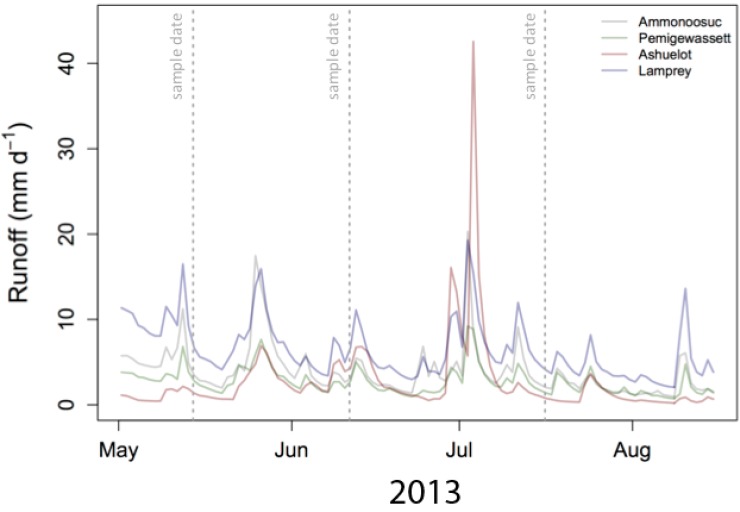
2013 hydrograph comparisons at four sites spanning our sampling domain. River discharge measurements from the Ammonoosuc (north central New Hampshire; USGS gage 01137500), Ashuelot (southwestern New Hampshire; USGS gage 01157000), Pemigewassett (central New Hampshire; USGS gage 01076500), and Lamprey (southeast New Hampshire; USGS gage 01064500). Dashed vertical lines indicate synoptic sampling events.

There was some hydrologic variability among sampling events (Fig 4). Mean daily runoff for four USGS gages spanning our sampling area was 3.6 mm/d in May (flows were elevated due to residual snow melt), 4.2 mm/d in June (collected between two storms), and 2.1 mm/d in July (low flow conditions).

### Analytical methods

Dissolved organic carbon was measured with a Shimadzu TOC analyzer, and major ions (Na, K, Mg, Ca, Cl) were measured on a Dionex X2 ion chromatograph at the University of New Hampshire in 2013. These water chemistry measurements were made using sub-samples from the polypropylene bottles that were filtered and held frozen prior to analysis. Metals and metalloid concentrations (hereafter named “metals”) were measured with an Inductively-Coupled Plasma-Mass Spectrometer (ICP-MS, Perkin Elmer NexION 300X) at the University of Pittsburgh in February 2014, 8–10 months after collection. Metal concentrations were determined from sub samples of the synoptic water isotope archive described above. Sub-samples for metals analysis from the glass vials were diluted with sub-boil distilled 2% nitric acid (HNO_3_) to standardize matrix composition and spiked with an internal standard of Be, Ge, and Tl. The ICP was calibrated with a five-point curve and blanks and drift checks measured roughly every 10 samples. For elements with strong polyatomic interferences (e.g., As) kinetic energy discrimination collision cell methods were used.

**Fig 5:**
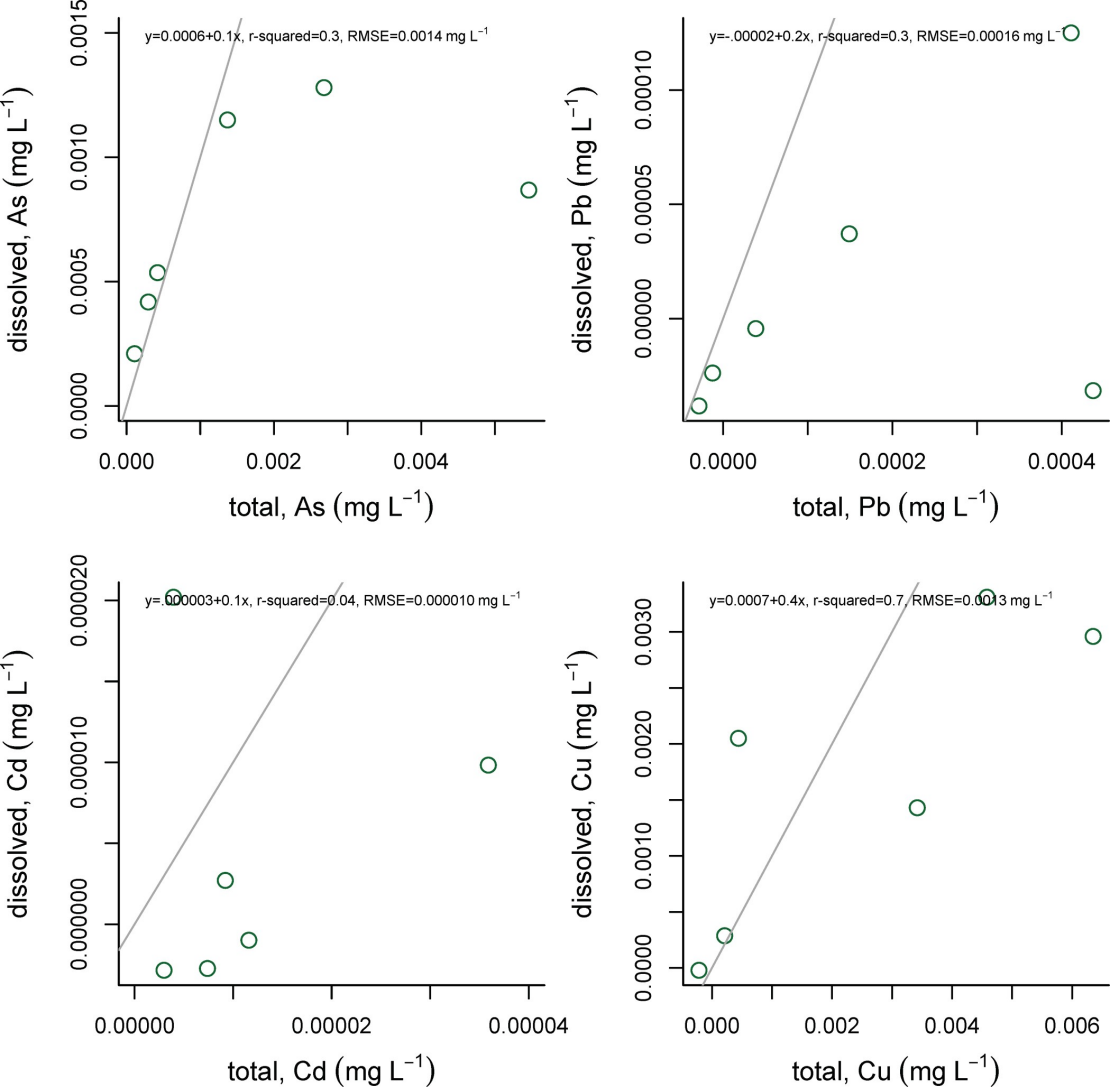
Comparison of metal concentration measurements on unfiltered, unacidified samples. Samples were stored in borosilicate glass (total; x-axes) and filtered, acidified samples stored in polypropylene vials (dissolved; y-axes). Zinc is not shown because it was not detectable in unfiltered and unacidified samples (e.g. bdc, hbk, hod, nwd, pbb, prp), and filtered and acid-preserved samples (e.g. hbk, hod, pbb, prp), and therefore comparisons could not be made between total and dissolved zinc concentrations.

These data were produced from a sample archive stored under conditions that do not follow typical conventions for dissolved metal preservation (samples were not filtered, not acidified, and stored in borosilicate vials). These samples should not be compared directly with either total or dissolved metal concentrations. To evaluate the impact of these storage conditions, a set of six samples were collected in July 2017 from sample sites that spanned the range of Na concentration and watershed land cover across our sampling domain, and collected and stored using both methods (filtered, acidified, polypropylene container and not filtered, not acidified, borosilicate glass container) (Fig 5). These samples were allowed to sit for four months to allow equilibration. Then metal concentrations in the two sets of samples were evaluated on the ICP-MS as described above and compared.

### Statistical methods

Data did not uniformly meet normality criteria and therefore relationships between trace metals and other biogeochemical parameters (e.g., DOC and Fe concentrations) were evaluated using Spearman’s rank order correlation with alpha = 0.05. All statistics were performed in R Computing Software [26].

## Results

Direct comparison of preservation methods demonstrates that the metal concentrations measured in the archived samples are not precisely comparable to the filtered and acidified samples (Fig 5). However, in general, the signal strengths are related positively, though substantial noise is introduced. Therefore, the measurements presented throughout this paper seem to reflect overarching trends in synoptic metal concentrations, albeit a relatively noisier version of that signal.

**Fig 6:**
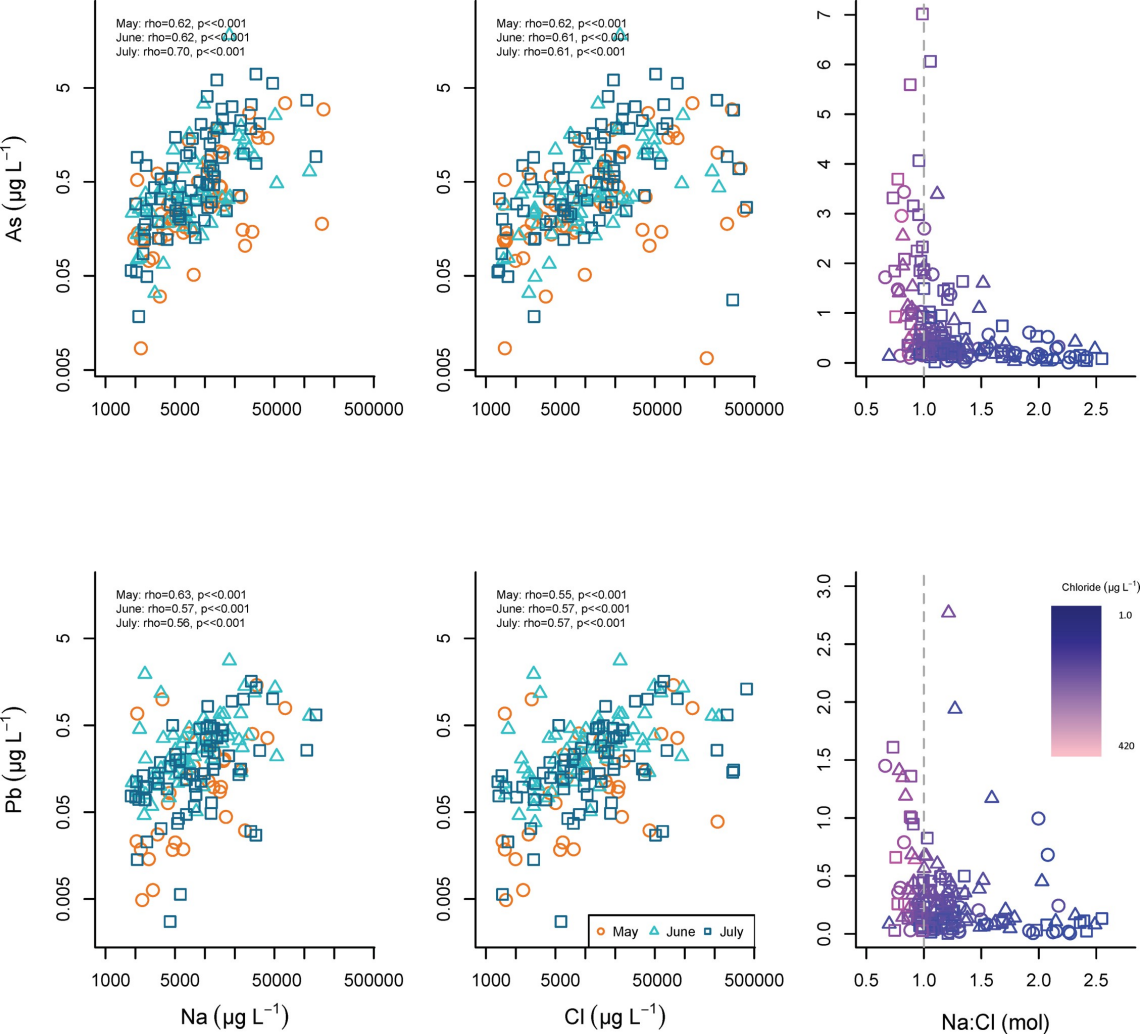
Arsenic and lead relationships with Na and Cl. Relationship between As and Pb concentrations and Na and Cl concentrations and Na: Cl molar ratios in synoptic samples collected May 14, June 11, and July 16, 2013. Na:Cl molar ratio is colored using a gradient from high Cl concentrations (light pink), to low Cl concentrations (dark purple).

**Fig 7:**
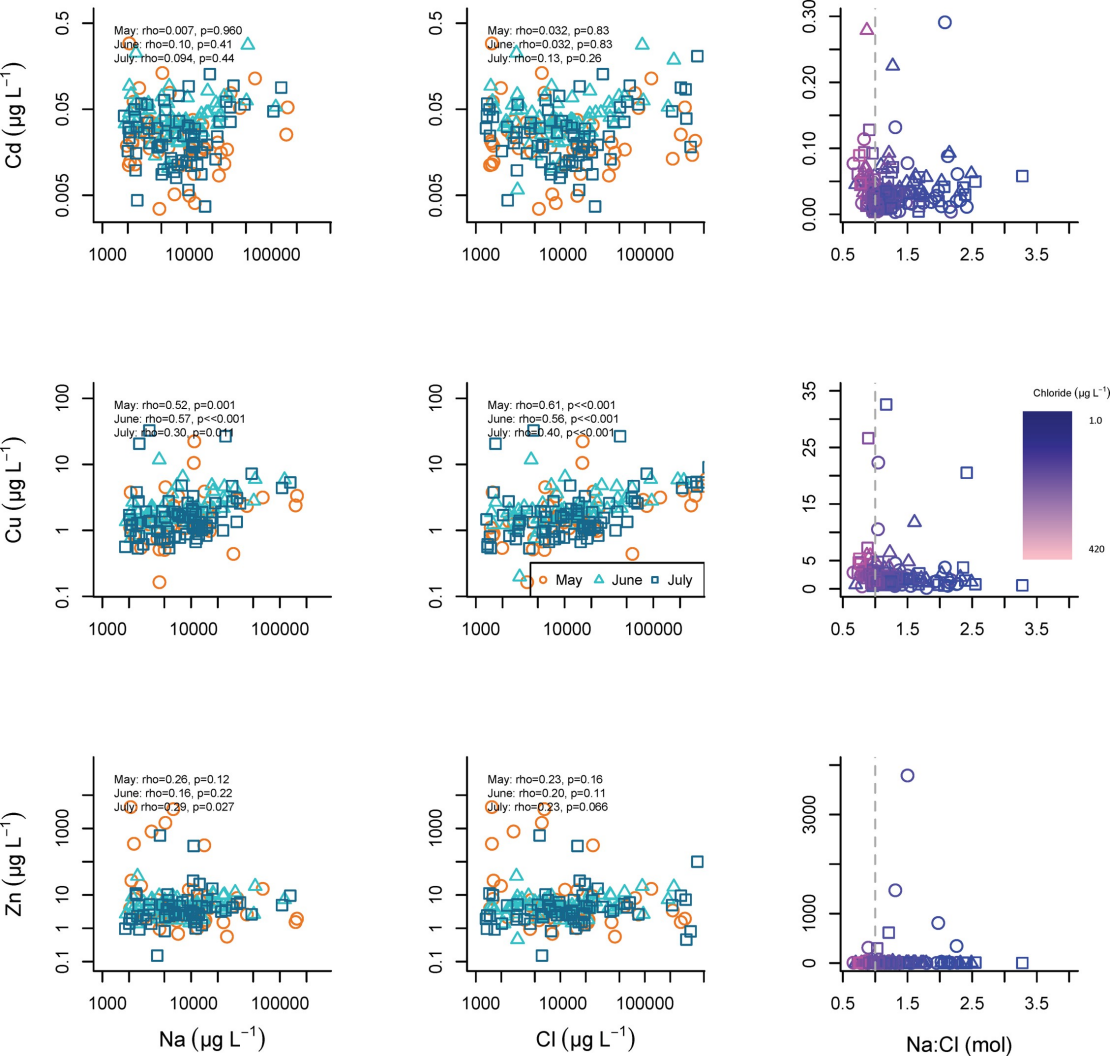
Cadmium, copper, and zinc relationships with Na and Cl. Relationships among Cd, Cu, and Zn concentrations and Na and Cl concentrations and Na: Cl molar ratios in synoptic samples collected May 14, June 11, and July 16, 2013. Na:Cl molar ratio is colored using a gradient from high Cl concentrations (light pink), to low Cl concentrations (dark purple).

**Fig 8:**
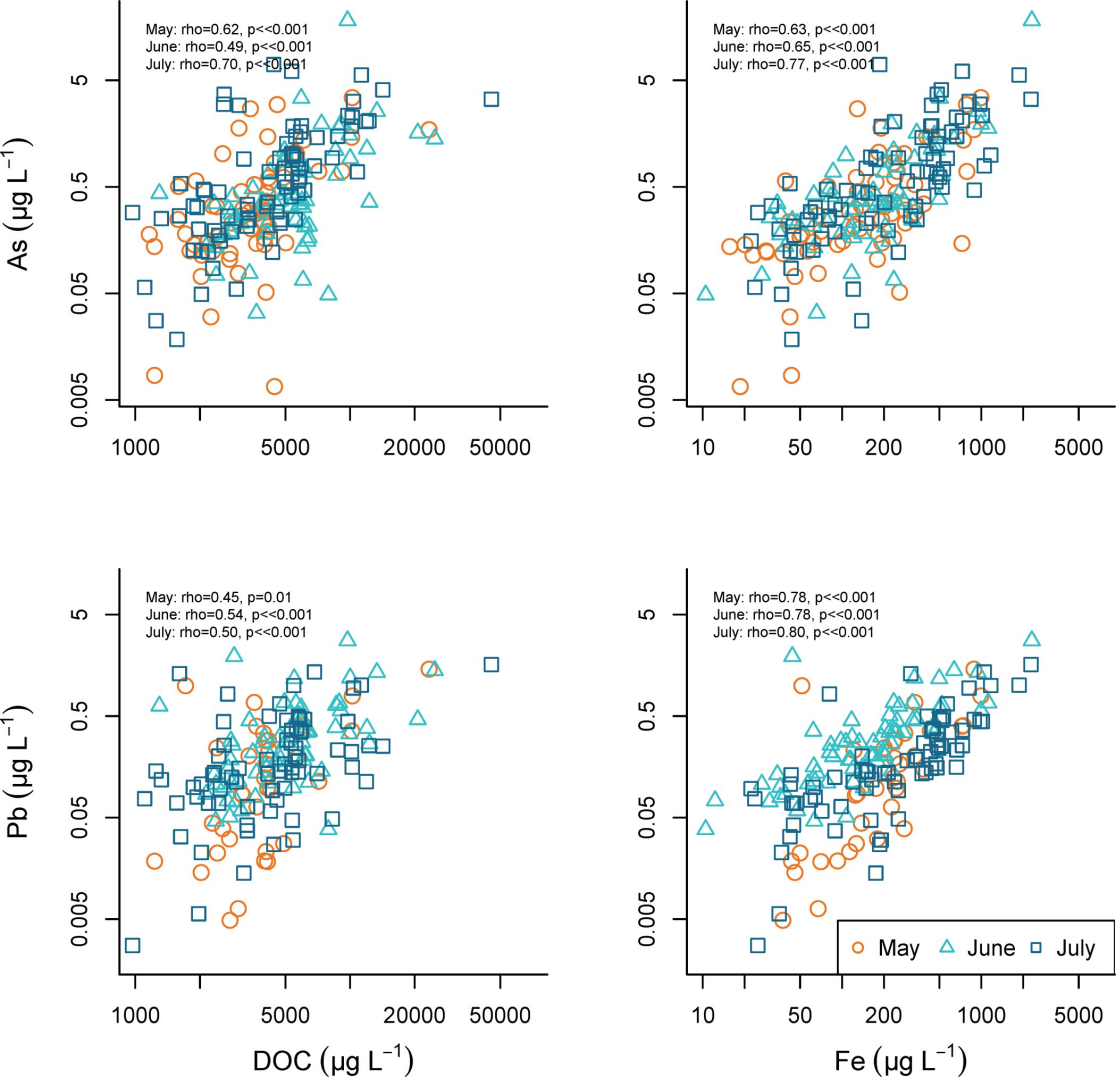
Arsenic and lead relationships with DOC and Fe. Relationships between As and Pb concentrations and dissolved organic carbon (DOC) and Fe concentrations during May 14, June 11, and July 16, 2013.

**Fig 9:**
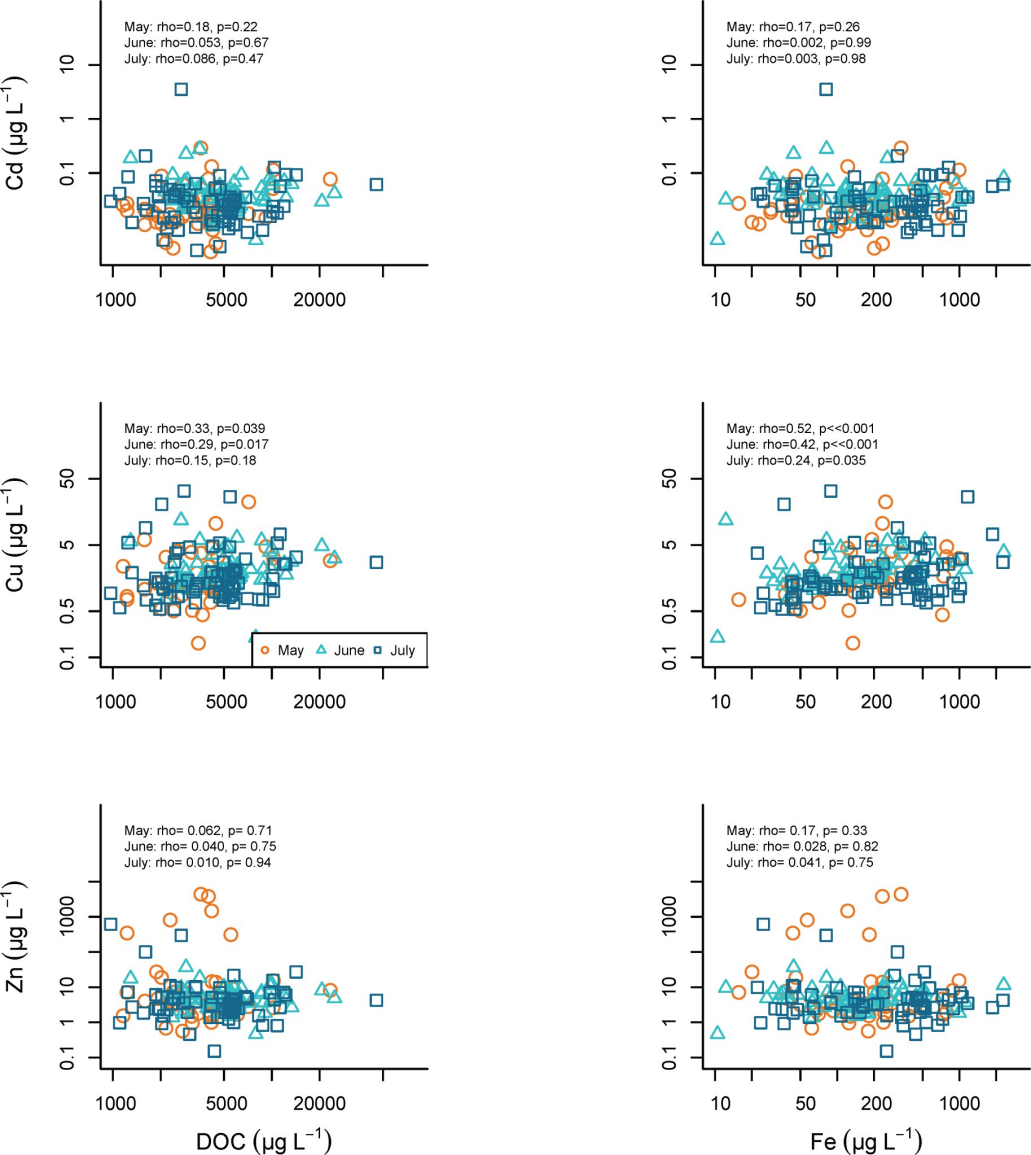
Cadmium, copper, and zinc relationships with DOC and Fe. Relationships among Cd, Cu, and Zn concentrations and dissolved organic carbon (DOC) and Fe concentrations during May 14, June 11, and July 16, 2013.

Quality assurance procedures (e.g., internal standard values) identified three of the synoptic samples that were clear outliers, and these samples were removed from all subsequent data analysis. In the remaining 191 samples, metal concentrations varied widely across the study area (Table 1). Despite the variability, median concentrations were relatively consistent across the three summer months. High concentrations of As and Pb (max As = 18.1 μg/L, max Pb = 2.77 μg/L) occurred in samples with elevated Cl and Na concentrations (Fig 6). Further, the highest concentrations of As, Cd, and Pb occurred in samples with molar Na: Cl ratios that approached 1.0 (Figs 6 and 7). Copper, Pb, and sometimes As concentrations were associated with samples containing elevated DOC concentrations (Figs 8 and 9). Samples with higher concentrations of As were associated with samples with higher concentrations of Fe.

**Table 1:** Monthly cation sample statistics.

Month	Samples (n)	Min (μg L^-1^)	25^th^ Percentile (μg L^-1^)	Med (μg L^-1^)	75^th^ Percentile (μg L^-1^)	Max (μg L^-1^)
**Arsenic**						
**May**	61	0.0067	0.15	0.28	0.61	3.4
**June**	63	0.033	0.22	0.35	0.73	18
**July**	77	0.019	0.25	0.5	1.5	7.02
**Cadmium**						
**May**	51	0.0035	0.014	0.0201	0.035	0.29
**June**	66	0.0058	0.031	0.038	0.055	0.28
**July**	73	0.0037	0.015	0.025	0.0407	3.5
**Copper**						
**May**	42	0.16	1.03	1.4	3.09	22
**June**	66	0.2	1.5	2	2.6	12
**July**	77	0.54	0.98	1.4	2.6	33
**Lead**						
**May**	35	0.0049	0.029	0.11	0.29	1.5
**June**	66	0.038	0.14	0.27	0.45	2.8
**July**	71	0.0028	0.078	0.14	0.35	1.6
**Zinc**						
**May**	41	0.56	1.8	3.9	14	4400
**June**	66	0.46	3.3	4.6	6.3	37
**July**	63	0.15	2.05	3.4	6	61

Monthly sample ranges of cation concentrations during May 14, June 11, and July 16, sampling. Site statistics include number of samples, minimum, 25^th^ percentile, median, 75^th^ percentile, and maximum cation concentrations.

### Elemental relationships

Metal concentration associations with DOC, Na and Cl, and Fe are consistent across months (Table 2). Over the course of the summer, arsenic and lead have the strongest relationships with all three indicators of chemical processes, i.e., cation exchange (Na, Cl) (Fig 6), dissolved organic carbon complexation (DOC), and oxidation-reduction reactions (Fe) (Fig 8).

**Table 2:** Evaluation of elemental relationships using Spearman’s rank order correlation.

Rho, n				
Month	Na	Cl	DOC	Fe
**Arsenic**				
**May**	*0*.*58*, 57	*0*.*52*, 61	*0*.*56*, 61	*0*.*65*, 61
**June**	*0*.*62*, 61	*0*.*61*, 63	*0*.*49*, 63	*0*.*65*, 63
**July**	*0*.*7*, 73	*0*.*61*, 77	*0*.*7*, 77	*0*.*77*, 77
**Cadmium**				
**May**	0.08, 47	0.099, 51	0.2, 51	0.18, 51
**June**	0.1, 64	0.16, 66	0.053, 66	0.002, 66
**July**	0.094, 69	0.13, 73	0.086, 73	0.003, 73
**Copper**				
**May**	0.39, 38	*0*.*5*, 42	0.34, 42	*0*.*5*, 42
**June**	*0*.*57*, 64	*0*.*56*, 66	0.29, 66	*0*.*42*, 66
**July**	0.3, 73	*0*.*4*, 77	0.15, 77	0.24, 77
**Lead**				
**May**	*0*.*5*, 31	0.43, 35	0.42, 35	*0*.*75*, 35
**June**	*0*.*57*, 64	*0*.*57*, 66	*0*.*54*, 66	*0*.*78*, 66
**July**	*0*.*56*, 67	*0*.*57*, 71	*0*.*5*, 71	*0*.*8*, 71
**Zinc**				
**May**	0.26, 37	0.23, 41	0.062, 41	0.17, 41
**June**	0.16, 64	0.2, 66	0.04, 66	0.028, 66
**July**	0.29, 59	0.23, 63	0.01, 63	0.041, 63
**DOC**				
**May**	0.4, 58	0.38, 62		*0*.*63*, 62
**June**	*0*.*57*, 64	*0*.*45*, 66		*0*.*72*, 66
**July**	*0*.*54*, 73	*0*.*46*, 77		*0*.*75*, 77
**Fe**				
**May**	*0*.*65*, 58	*0*.*59*, 62	*0*.*63*, 62	
**June**	*0*.*67*, 64	*0*.*67*, 66	*0*.*72*, 66	
**July**	*0*.*7*, 73	*0*.*67*, 77	*0*.*75*, 77	

Monthly assessment of elemental relationships during May 14, June 11, and July 16 sampling. Italicized values indicate significance *(p<<0*.*001)*. Site statistics include rho, p values, and sample count (n). Smaller sample sizes in May for copper, lead, and zinc are due to concentrations below detection limits.

#### Arsenic

Arsenic relationships with other metals and DOC are relatively consistent across months, and the strongest associations are observed in July (Figs 6 and 8, Table 2). Arsenic is strongly related to Na and Cl throughout the summer, and the highest concentrations of As occur as the molar Na: Cl ratio approaches one, the characteristic value of halite (Fig 6).

#### Cadmium

Cadmium has no apparent relationships with Fe, DOC (Fig 8), nor with Na and Cl concentrations (Fig 7). Elevated cadmium concentrations occur as the Na: Cl ratio approaches one (Fig 7).

#### Copper

Copper relationships with DOC and Fe are similar across months (Fig 9), and Cu concentrations are associated with Na and Cl (Fig 7). Copper concentrations are elevated with Na: Cl ratio values near 1.

#### Lead

Lead is associated with DOC and Fe throughout the summer months (Fig 8), and Na and Cl concentrations in May, June, and July (Fig 6). Lead concentrations are highest in samples with Na: Cl ratios that approach the 1.0 molar ratio.

#### Zinc

Zinc does not seem to correlate with Fe or DOC (Fig 9). Zinc concentrations are not related to Na or Cl concentrations (Fig 7).

#### Dissolved organic carbon, iron, sodium chloride

**Fig 10:**
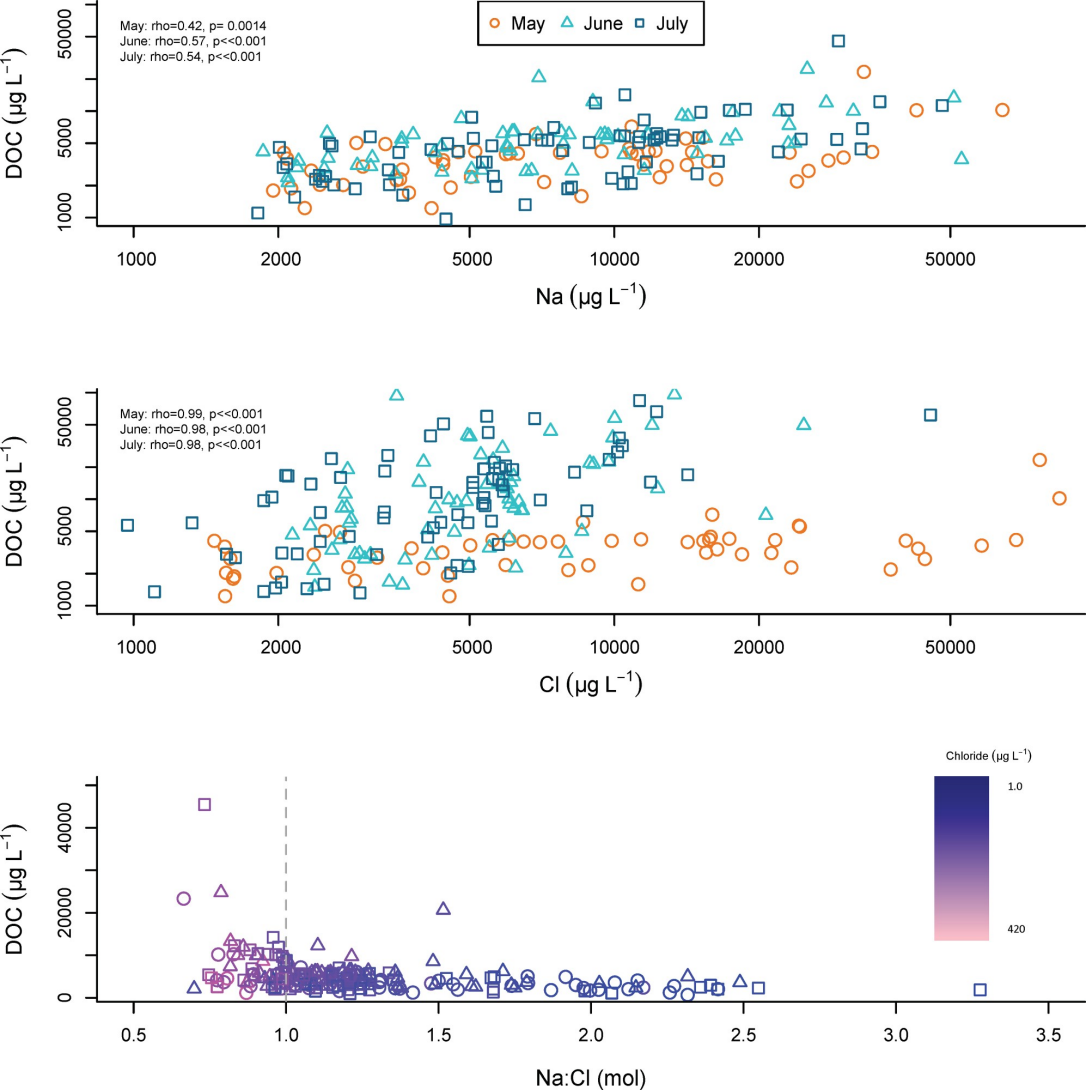
Na and Cl relationships with DOC. Concentrations of Na, Cl, and Na: Cl molar ratio plotted against dissolved organic carbon (DOC) concentrations for all locations throughout northern New England on May 14, June 11, and July 16, 2013. Na:Cl molar ratio is colored using a gradient from high Cl concentrations (light pink), to low Cl concentrations (dark purple).

Dissolved organic carbon is associated with Na throughout the summer months, and with Cl (Fig 10). Slight increases in DOC concentrations occur near 1.0 Na: Cl. Dissolved organic carbon is strongly correlated with Fe throughout May, June, and July. Iron is strongly associated with Na and Cl.

## Discussion

It is difficult to directly compare our water chemistry measurements with dissolved concentration data commonly reported in the literature. In this study, trace metal concentrations were measured in subsamples of an unfiltered, unacidified archive that was stored in borosilicate containers. This raises the potential for 1) the measurement of metals bound to particles (i.e., larger than 0.45 micron) that would be removed by filtration; 2) sorption of dissolved metals to the borosilicate surfaces; and 3) chemical precipitation processes and the formation of particulates that sorb dissolved trace metals (e.g., iron oxidation and the formation of iron hydroxides). Our direct comparison of samples subject to both the conditions for the synoptic sample archive and a standard dissolved metal measurement suggest that a mixture of these potential processes likely occurred, with concentrations in the synoptic archive measuring both above and below the concentrations measured in “dissolved metal” samples. Nonetheless, the measurement of the archived samples captures interesting patterns across broad spatial scales; although these patterns are likely more noisy than those that would be observed under optimal preservation methods, the limitations of archive conditions do not preclude assessment of spatial trends in metals.

Our data are consistent with road salt mobilization of trace metals and the resultant elevated streamwater concentrations of these metals. Observed Na and Cl concentrations range from highly dilute (min Na = 1800 μg/L; min Cl = 1320 μg/L) to highly concentrated (max Na = 160000 μg/L; max Cl = 420000 μg/L). The strong, positive correlation between trace metal concentrations (Pb, As, Cu, and Zn) and both Na and Cl indicate trace metal concentrations are elevated in salty waters. Further, the elevated concentrations of Pb, As, and Cd in samples where dissolved Na: Cl ratios are near 1:1 (i.e., the ratio in halite) provide a parallel and consistent line of evidence that road salt mobilizes trace metals. The Na:Cl ratio can vary in surface waters both above 1 (during periods after salting when lagged Na transport occurs) and below 1 (during the early salt flux where Na is sorbed and Cl is transported rapidly). In addition, other common sources of Na and Cl are not 1:1. For example, rainfall in experimental catchments is ~0.8 [27,28]. We do not have the data to claim causality, but the clear tendency warrants continued attention. While trace metals are mobilized, the concentrations are relatively low, and streamwater metal concentrations were always low relative to US EPA drinking water standards (Table 3). Some sites with a strong road salt signature had metal concentrations approaching levels of concern with concentrations above EPA freshwater chronic criteria for aquatic organisms measured at several stations. Comparisons of our measured trace metal values with water quality standards should be made cautiously due to the archival nature of the samples.

**Table 3:** USEPA national water quality criteria [29].

Element	Drinking Water Maximum Contaminant Level (MCL) (μg L^-1^)	Aquatic Life Chronic Criteria (μg L^-1^)	Aquatic Life Acute Criteria (μg L^-1^)
As	10	150	340
Cd	5	0.72	1.8
Cu	1300	^a^Not shown	
Pb	15	2.5	65
Zn	-	120	120

^a^Site-specific calculations needed for ligand model criteria.

Previous studies of trace metal mobilization to surface waters by road salt have focused on limited spatial scales. Bäckström et al [16] studied two transects located at most 30m from the roadway, Löfgren [20] studied five small forested catchments (drainage areas 57–168 hectares), and Cunningham et al. [30] collected samples within the limits of a single upstate NY city (76 hectare college campus and one additional off campus site). Our sites span tens of thousands of km^2^, and allow evaluation of the road salt signal at much broader scales. Across these sites, and during each month, trace metals were consistently related to both Na and Cl. We interpret the strength of these relationships across a large scale, and their persistence across months, to indicate a general mechanism or set of mechanisms related to road salt driven salinization. Our data cannot reveal the patterns of hydrologic transport that would move these trace metals from soil pores to stream water. However, these mechanisms seem to alter trace metal concentrations regardless of heterogeneity in hydrologic transport processes across wide spatial scales.

Further, our data indicate that trace metals are mobilized by processes that parallel or interact with road salt chemical fluxes, processes that are consistent across broad spatial scales. Most importantly, As and Pb are related to both Fe and DOC concentrations across all months sampled. These associations suggest a number of potential mechanisms. First, Mn and Fe oxides in soils and sediments strongly sorb many cations [31]. In particular, As has strong adsorption affinities for reduced Fe species, Fe (III) oxyhydroxides, and can be released to groundwater during Fe-reducing biodegradation [32]. When these oxides are reduced, any sorbed cations are mobilized [33,34]. Therefore, any flushing of reducing zones by waters rich in road salt could mobilize dissolved metals in those waters. Alternatively, the association between Pb/As and DOC suggests that road salt chemical fluxes can enhance the DOC flux and therefore metals associated with the DOC. For example, Amrhein et al. [15,18] observed NaCl mobilizes metals associated with organic matter and colloids. However, ultimately, the covariance of Fe and DOC suggests increases in Fe could also simply result from increased amounts of DOC-sorbed Fe during increased DOC flux that arises from salting. Increase in soil salinity can cause DOC to flocculate with cations (Ca^2+^ and Mg^2+^) [35], enhancing metal mobility from soils to streams. Therefore, the NaCl interactions with DOC can potentially explain both associations (Pb/As with Fe and Pb/As with DOC) and reduction/oxidation dynamics may not be an important control. Our results do not allow us to partition the relative importance of salt pulses, redox conditions, and organic carbon to metal mobilization, though the consistent associations among salt, Pb/As, DOC, and Fe suggests these interactions should be more closely examined as inquiry into road salt effects continues.

It also should be noted that elevated As has been documented in groundwaters across the southern part of our sampling domain. These concentrations are attributed partially to naturally occurring As associated with weathering of bedrock minerals [36,37]. While our data demonstrate a relationship with road salt indicators, we cannot quantify the potential contribution of naturally occurring As. This is particularly challenging as areas rich in natural As sources are also the most urban. Deicer impacts covary spatially with bedrock As hotspots and observed associations could result from this covariance. However, the other trace metals we measured (Pb, Cu, Zn, and Cd) do not have major, documented natural sources in this region. Given the consistency in the observed relationships between these metals and deicer impacts, this covariance driving the As signal alone seems less likely. Potential anthropogenic sources of Pb, Cu, Zn, Cd, and As include road materials and mobile source emissions [38] and general trace metal pollution sources [37,38]. In particular, As and Pb enrichment associated with historical use of lead arsenate as a pesticide in orchards has been observed in study area streamwaters [39]. The bottom line is that there are many potential sources of trace metal contamination, however, road deicers and associated chemistry seem to mobilize the metals regardless of source.

## Conclusions

The patterns in our data highlight several important aspects of surface water salinization. Persistent patterns in stream water chemistry across broad spatial scales suggest New England flowing waters are influenced by pools with elevated Na and Cl concentrations (e.g., soil and ground water). Soils, where most of the mechanistic work on metals mobilization has been conducted, are only ephemerally connected hydrologically to streams—primarily during storm events. In contrast, groundwater contributes a substantial fraction of streamwater flow, especially during low flows (i.e., our sampled conditions). Detailed hillslope studies to trace the movement of mobilized trace metals in soils [e.g., 21], and to determine whether they are laterally transported to drainage systems or streams, or vertically percolated into groundwater can clarify the relative importance of the pool. Second, the observed broad spatial interactions among NaCl, trace metals, Fe, and DOC indicate that documentation of road salt impacts should examine a broader range of chemical parameters. Without a broader perspective, mechanism can be misattributed, and therefore, mitigation based on potential misunderstandings will be less effective.

## Supporting information

S1 File **Table A. Streamwater and river collected ion site-specific information.** Water sample data collected across northern New England and analyzed for metals and water quality; sites marked with (*) include those samples removed from subsequent statistical analysis. Samples indicated as ‘bdl’ are due to concentrations below detection limits. **Table B. Land use and landscape site-specific information.** Site characteristics across northern New England from the New Hampshire Land Cover archive (NLCD 2011); sites marked with (*) include those samples removed from subsequent statistical analysis. **Table C. Contrast dissolved and total sample site-specific information.** Resampled and analyzed ion data comprising a subset of our sites in northern New England. Samples indicated as ‘bdl’ are due to concentrations below detection limits.
(DOC)
