# Supplementary material for: Trace metals in Northern New England streams: Evaluating the role of road salt across broad spatial scales with synoptic snapshots
Source: PLoS One. 2019 Feb 13;14(2):e0212011. doi: 10.1371/journal.pone.0212011 (PMC6373959; doi:10.1371/journal.pone.0212011)
Supplement: S1 File — Table A. Streamwater and river collected ion site-specific information. Water sample data collected across northern New England and analyzed for metals and water quality; sites marked with (*) include those samples removed from subsequent statistical analysis. Samples indicated as ‘bdl’ are due to concentrations below detection limits. Table B. Land use and landscape site-specific information. Site characteristics across northern New England from the New Hampshire Land Cover archive (NLCD 2011); sites marked with (*) include those samples removed from subsequent statistical analysis. Table C. Contrast dissolved and total sample site-specific information. Resampled and analyzed ion data comprising a subset of our sites in northern New England. Samples indicated as ‘bdl’ are due to concentrations below detection limits. (DOC) [file pone.0212011.s001.doc]

Supporting Information

Trace metals in Northern New England streams: Evaluating the role of road salt across broad spatial scales with synoptic snapshots

Jessica F. Wilhelm^1^, Daniel J. Bain^2^, Mark B. Green^1,3^, Kathleen F. Bush^1,4^, William H. McDowell^5^

^1^ Center for the Environment, Plymouth State University, Plymouth, New Hampshire, United States of America

^2^ Department of Geology and Environmental Science, University of Pittsburgh, Pittsburgh, Pennsylvania, United States of America

^3^ Northern Research Station, United States Forest Service, Durham, New Hampshire, United States of America

^4^ New Hampshire Department of Health and Human Services, Concord, New Hampshire, United States of America

^5^ Natural Resources and the Environment, University of New Hampshire, Durham, New Hampshire, United States of America

**Table A. Streamwater and river collected ion site-specific information.** Water sample data collected across northern New England and analyzed for metals and water quality; sites marked with (*) include those samples removed from subsequent statistical analysis. Samples indicated as ‘bdl’ are due to concentrations below detection limits.

| Site ID | Date | Latitude | Longitude | River | Sodium, µg/L | Iron, µg/L | Copper, µg/L | Zinc, µg/L | Arsenic, µg/L | Cadmium, µg/L | Lead, µg/L | DOC, µg/L | Chloride, µg/L |
| --- | --- | --- | --- | --- | --- | --- | --- | --- | --- | --- | --- | --- | --- |
| ARD 5 | 5/14/13 | 44.460618 | -71.186188 | Androscoggin | 3340 | 127 | 1.24 | bdl | 0.612 | 0.025 | 0.028 | 4906 | 2687 |
| ARU 5 | 5/14/13 | 44.503668 | -71.156587 | Androscoggin | 2907 | 193 | bdl | bdl | 0.149 | bdl | bdl | 5023 | 2502 |
| BAT 5 | 5/14/13 | 43.070944 | -70.783111 | Borthwick Ave | bdl | 788 | 4.71 | bdl | 0.697 | 0.015 | bdl | 9171 | 364156 |
| BBD 5 | 5/14/13 | 42.923317 | -72.2726 | Beaver Brook | 29980 | 726 | 0.440 | 4.61 | 0.147 | 0.017 | 0.397 | 3668 | 58096 |
| BBK 5 | 5/14/13 | 42.907736 | -71.922104 | Bogle Brook | 4405 | 125 | 0.513 | 0.955 | 0.308 | 0.032 | 0.083 | 3165 | 4388 |
| BBU 5 | 5/14/13 | 42.957194 | -72.266417 | Beaver Brook | 15665 | 333 | 1.88 | bdl | 0.286 | 0.018 | 0.203 | 3382 | 16369 |
| BBW 5 | 5/14/13 | 42.930722 | -72.271444 | Beaver Brook | 23146 | 386 | bdl | 1.52 | 0.345 | 0.021 | 0.150 | 4075 | 40396 |
| BDU 5 | 5/14/13 | 43.546528 | -72.270667 | Blood Brook | 8542 | 79.1 | 1.07 | 4.02 | 0.505 | 0.011 | bdl | 1590 | 11212 |
| BLD 5 | 5/14/13 | 45.050556 | -71.385694 | Back Lake Brook | 6057 | 93.0 | 1.61 | 2.11 | 0.144 | 0.018 | 0.019 | 4000 | 7634 |
| BLU 5 | 5/14/13 | 45.052389 | -71.383472 | Back Lake Brook | 4738 | 70.5 | 0.679 | 1.75 | 0.191 | 0.003 | 0.018 | 4123 | 5581 |
| BRD 5 | 5/14/13 | 43.1795 | -70.887 | Bellamy River | 14083 | 183 | bdl | 311.03 | 1.05 | bdl | bdl | 5528 | 24316 |
| BRU 5 | 5/14/13 | 43.178861 | -70.890778 | Bellamy River | 15290 | 238 | 2.17 | 9.14 | 1.02 | 0.033 | 0.194 | 5631 | 24201 |
| BSW 5 | 5/14/13 | 42.924361 | -72.270389 | Baker St. Storm Water | 150417 | 182 | 2.39 | 1.54 | 0.179 | 0.025 | bdl | 1162 | 266758 |
| BWS 5 | 5/14/13 | 44.060472 | -71.292917 | Louisville Brook | 1951 | 28.5 | bdl | bdl | 0.125 | 0.019 | bdl | 1797 | 1610 |
| CBT 5 | 5/14/13 | 43.771667 | -71.72989 | Clay Brook | 6299 | 234 | 2.36 | 3789.79 | 0.164 | 0.078 | 0.123 | 3997 | 6475 |
| CBU 5 | 5/14/13 | 43.7454 | -71.719 | Clay Brook | 3623 | 285 | bdl | bdl | 0.229 | 0.018 | bdl | 2824 | 3212 |
| CCK 5 | 5/14/13 | 42.7692 | -70.9165 | Cart Creek | 64162 | 993 | 3.15 | 15.31 | 3.44 | 0.114 | 0.790 | 10238 | 119561 |
| CRD 5 | 5/14/13 | 43.800917 | -71.437472 | Creamery Brook | 25346 | 179 | bdl | 0.562 | 0.105 | 0.016 | 0.031 | 2747 | 44260 |
| CRU 5 | 5/14/13 | 43.803889 | -71.442278 | Creamery Brook | 23989 | 102 | bdl | bdl | 0.154 | 0.008 | bdl | 2189 | 37579 |
| CSP 5 | 5/14/13 | 42.5495 | -71.0909 | Cedar Swamp | 33047 | 885 | 2.90 | 8.13 | 1.72 | 0.077 | 1.45 | 23325 | 76732 |
| DCF 5 | 5/14/13 | 43.13483 | -71.18219 | Dowst-Cate | 6870 | 742 | 1.69 | 1.67 | 1.37 | 0.035 | 0.403 | 6081 | 8608 |
| DGB 5 | 5/14/13 | 44.016969 | -71.322302 | Douglas Brook | 2729 | 45.9 | 0.542 | 18.78 | 0.072 | 0.088 | 0.014 | 2026 | 1983 |
| EMB 5 | 5/14/13 | 44.737083 | -71.439722 | Emerson Brook | 2268 | 28.8 | bdl | bdl | 0.122 | 0.021 | bdl | 674 | 1510 |
| HOB 5 | 5/14/13 | 43.082111 | -70.796 | Hodgson Brook | 156036 | 782 | 3.34 | 1.94 | 2.96 | 0.053 | bdl | 4578 | 298315 |
| IDM 5 | 5/14/13 | 42.6597 | -70.8942 | Ipswich Dam | 42522 | 430 | 2.34 | 2.59 | 1.46 | 0.052 | 0.357 | 10175 | 84342 |
| IRD 5 | 5/14/13 | 44.411635 | -71.496001 | Israel River | 4402 | 134 | 0.164 | 1.80 | 0.203 | bdl | bdl | 3457 | 3785 |
| IRU 5 | 5/14/13 | 44.361415 | -71.397224 | Israel River | 2997 | 67.4 | 1.37 | 2.16 | 0.077 | 0.028 | 0.006 | 3015 | 2370 |
| JOB 5 | 5/14/13 | 44.758194 | -71.427028 | Johnson Brook | 2270 | 43.4 | 0.838 | 345.01 | 0.009 | 0.020 | 0.019 | 1230 | 1550 |
| MOD 5 | 5/14/13 | 44.373481 | -71.296647 | Moose River | 4566 | 39.3 | 1.32 | 4.69 | 0.568 | 0.016 | bdl | 1917 | 4506 |
| MOU 5 | 5/14/13 | 44.373392 | -71.298639 | Moose River | 7143 | 61.9 | 3.28 | 0.681 | 0.151 | 0.005 | bdl | 2159 | 8024 |
| MRC 5 | 5/14/13 | 43.940361 | -71.511194 | Mad River | 3524 | 42.3 | bdl | bdl | 0.030 | 0.035 | bdl | 2249 | 4002 |
| MRL 5 | 5/14/13 | 43.969111 | -71.50725 | Mad River | 2341 | 37.6 | 0.893 | 7.61 | 0.118 | 0.061 | 0.005 | 2761 | 1589 |
| NEA 5 | 5/14/13 | 43.176972 | -71.831222 | Contoocook | 11150 | 282 | 1.46 | bdl | 0.303 | 0.017 | 0.339 | 3956 | 14208 |
| NEB 5 | 5/14/13 | 43.178361 | -71.822028 | Contoocook | 10854 | 235 | 1.41 | 3.05 | 0.366 | bdl | 0.286 | 4056 | 15319 |
| NED 5 | 5/14/13 | 43.17375 | -71.816833 | Contoocook | 11791 | 239 | 1.21 | 1.00 | 0.607 | 0.038 | 0.288 | 4172 | 15749 |
| NEU 5 | 5/14/13 | 43.173472 | -71.817472 | Contoocook | 12433 | 201 | 1.56 | 7.22 | 0.325 | 0.004 | 0.243 | 2396 | 8830 |
| NSB 5 | 5/14/13 | 42.740444 | -71.471861 | Salmon Brook | 34377 | 405 | bdl | bdl | 1.47 | bdl | bdl | 4139 | 68525 |
| NWD 5 | 5/14/13 | 43.081194 | -70.797917 | Newfields Ditch | bdl | 279 | 4.02 | 3.44 | 1.02 | 0.013 | 0.039 | 2553 | 213919 |
| OBG 5 | 5/14/13 | 42.917456 | -71.916053 | Otter Brook | 10864 | 249 | 22.32 | 2.80 | 0.699 | 0.018 | 0.114 | 7154 | 15985 |
| OSS 5 | 5/14/13 | 43.79239 | -70.99248 | Ossipee River | 5030 | 50.0 | 0.505 | 3.15 | 0.169 | bdl | 0.022 | 2413 | 5941 |
| PBB 5 | 5/14/13 | 43.994875 | -71.352244 | Pine Bend Brook | 2126 | 20.1 | bdl | 27.13 | 0.143 | 0.01 | bdl | 1884 | 1619 |
| PBD 5 | 5/14/13 | 42.969694 | -71.697861 | Piscataquog | 10731 | 235 | 10.53 | 13.84 | 0.840 | 0.00 | bdl | 4446 | 15864 |
| PBI 5 | 5/14/13 | 42.978833 | -71.473139 | Piscataquog | 14153 | 129 | 3.88 | 1.48 | 0.451 | bdl | 0.086 | 3122 | 21214 |
| PBU 5 | 5/14/13 | 42.979889 | -71.689139 | Piscataquog | 9403 | 189 | bdl | 14.41 | bdl | 0.012 | bdl | 4199 | 11365 |
| PIN 5 | 5/14/13 | 43.76196 | -71.13488 | Pine River | 7708 | 259 | 1.32 | bdl | 0.051 | 0.026 | 0.169 | 4064 | 9874 |
| PRF 5 | 5/14/13 | 43.437833 | -71.652333 | Pemigewasset | 4245 | 227 | bdl | bdl | 0.282 | 0.034 | 0.064 | 3686 | 5015 |
| PRG 5 | 5/14/13 | 43.017306 | -71.601167 | Piscataquog (Main) | 12176 | 175 | 1.03 | 3.40 | 0.553 | 0.009 | 0.097 | 4251 | 17347 |
| PWU 5 | 5/14/13 | 43.109028 | -71.762039 | Piscataquog (North) | 16270 | 138 | 0.977 | 4.04 | 0.331 | 0.017 | 0.044 | 2286 | 23338 |
| SBK 5* | 5/14/13 | 42.5236 | -71.185 | Sawmill Brook | bdl | 18.6 | bdl | bdl | 0.007 | bdl | bdl | 4450 | 166621 |
| SBM 5 | 5/14/13 | 43.170561 | -71.217811 | Saddleback | 4166 | 15.6 | 0.749 | 7.10 | 0.138 | 0.027 | bdl | 1228 | 4538 |
| SCD 5 | 5/14/13 | 44.88275 | -71.071956 | Dead Diamond River | 2055 | 113 | 1.10 | 3.90 | 0.291 | 0.011 | 0.023 | 4062 | 1472 |
| SCS 5* | 5/14/13 | 44.881867 | -71.072094 | Swift Diamond River | 2085 | 333 | 3.77 | 4378.50 | 0.525 | 0.291 | 0.682 | 3581 | 1547 |
| SHB 5 | 5/14/13 | 42.954694 | -71.793389 | School House Brook | 3738 | 51.5 | bdl | bdl | 0.180 | bdl | 0.995 | 1714 | 2887 |
| SLB 5 | 5/14/13 | 44.665389 | -71.457417 | Slide Brook | 2439 | 22.9 | bdl | bdl | 0.114 | 0.011 | bdl | 2036 | 1557 |
| SQB 5 | 5/14/13 | 43.703639 | -71.501333 | Unnamed | 11513 | 66.8 | bdl | bdl | 0.270 | bdl | bdl | 3152 | 15522 |
| SQM 5 | 5/14/13 | 43.723806 | -71.559556 | Mill Brook | 5949 | 41.7 | 1.16 | bdl | 0.234 | 0.027 | bdl | 3946 | 7001 |
| SRM 5 | 5/14/13 | 43.988306 | -71.327444 | Swift River | 3583 | 56.9 | bdl | 809.45 | 0.125 | 0.037 | bdl | 2298 | 2797 |
| SRN 5 | 5/14/13 | 43.391472 | -72.186306 | Sugar River | 14508 | 245 | 1.03 | 1.77 | 0.436 | 0.016 | 0.098 | 4127 | 21605 |
| SRU 5* | 5/14/13 | 44.023588 | -71.435663 | Swift River | 5137 | 122 | 4.51 | 1470.52 | 0.212 | 0.132 | 0.230 | 4187 | 6041 |
| SWP 5 | 5/14/13 | 43.756861 | -71.68725 | Storm Drain | bdl | 192 | 6.03 | bdl | 0.247 | 0.022 | bdl | 1582 | 395565 |
| WBG 5 | 5/14/13 | 43.0165 | -71.60125 | Piscataquog (Main) | 12851 | 191 | bdl | bdl | 1.78 | bdl | bdl | 3038 | 18429 |
| WHB 5 | 5/14/13 | 43.122344 | -71.004961 | Wednesday Hill Brook | 27883 | 129 | bdl | bdl | 2.70 | 0.012 | bdl | 3434 | 42854 |
| ARU 6 | 6/11/13 | 44.503668 | -71.156587 | Androscoggin | 2526 | 161 | 1.84 | 2.36 | 0.371 | 0.036 | 0.206 | 6234 | 2277 |
| BAT 6 | 6/11/13 | 43.070944 | -70.783111 | Borthwick Ave | 112910 | 523 | 5.87 | 7.30 | 0.642 | 0.053 | 0.641 | 8620 | 188579 |
| BBD 6 | 6/11/13 | 42.923317 | -72.2726 | Beaver Brook | 17869 | 248 | 2.10 | 10.00 | 0.344 | 0.046 | 0.448 | 5853 | 30198 |
| BBK 6 | 6/11/13 | 42.907736 | -71.922104 | Bogle Brook | 3612 | 499 | 2.28 | 7.61 | 0.383 | 0.050 | 1.170 | 5496 | 3500 |
| BBU 6 | 6/11/13 | 42.957194 | -72.266417 | Beaver Brook | 11767 | 277 | 2.10 | 3.96 | 0.369 | 0.023 | 0.344 | 5967 | 16732 |
| BBW 6 | 6/11/13 | 42.930722 | -72.271444 | Beaver Brook | 15465 | 320 | 2.70 | 5.35 | 0.244 | 0.027 | 0.671 | 5597 | 23363 |
| BDC 6 | 6/11/13 | 43.094303 | -70.987578 | Burley Demerrit | 6966 | 344 | 4.81 | 8.11 | 1.60 | 0.030 | 0.463 | 20662 | 7088 |
| BLD 6 | 6/11/13 | 45.050556 | -71.385694 | Back Lake Brook | 5996 | 179 | 2.10 | 1.78 | 0.341 | 0.012 | 0.179 | 4527 | 9941 |
| BLU 6 | 6/11/13 | 45.052389 | -71.383472 | Back Lake Brook | 4366 | 168 | 0.937 | 1.91 | 0.459 | 0.033 | 0.203 | 4649 | 4929 |
| BRD 6 | 6/11/13 | 43.1795 | -70.887 | Bellamy River | 14232 | 1120 | 2.16 | 3.69 | 1.78 | 0.038 | 0.676 | 8892 | 21834 |
| BRU 6 | 6/11/13 | 43.178861 | -70.890778 | Bellamy River | 13826 | 994 | 1.53 | 1.85 | 1.94 | 0.030 | 0.566 | 9048 | 21269 |
| BSW 6 | 6/11/13 | 42.924361 | -72.270389 | Baker St. Storm Water | bdl | 257 | 5.73 | 17.52 | 0.434 | 0.187 | 0.629 | 1295 | 220732 |
| BWS 6 | 6/11/13 | 44.060472 | -71.292917 | Louisville Brook | 2096 | 39 | 0.757 | 4.63 | 0.138 | 0.045 | 0.085 | 2139 | 4628 |
| CBT 6 | 6/11/13 | 43.771667 | -71.72989 | Clay Brook | 5863 | 204 | 1.54 | 2.97 | 0.210 | 0.022 | 0.203 | 6185 | 8253 |
| CBU 6 | 6/11/13 | 43.7454 | -71.719 | Clay Brook | 3815 | 236 | 2.05 | 5.04 | 0.067 | 0.019 | 0.355 | 6040 | 4332 |
| CCK 6 | 6/11/13 | 42.7692 | -70.9165 | Cart Creek | 50844 | 942 | 2.79 | 2.66 | 2.55 | 0.063 | 1.35 | 13335 | 95895 |
| CRD 6 | 6/11/13 | 43.800917 | -71.437472 | Creamery Brook | 17143 | 204 | 4.79 | 6.29 | 0.326 | 0.046 | 0.379 | 5274 | 26194 |
| CRU 6 | 6/11/13 | 43.803889 | -71.442278 | Creamery Brook | 14883 | 191 | 1.86 | 4.11 | 0.338 | 0.044 | 0.312 | 4009 | 22363 |
| CSP 6 | 6/11/13 | 42.5495 | -71.0909 | Cedar Swamp | 25190 | 638 | 3.14 | 4.84 | 1.41 | 0.042 | 1.41 | 24781 | 49386 |
| DCF 6 | 6/11/13 | 43.13483 | -71.18219 | Dowst-Cate | 4803 | 481 | 1.49 | 6.27 | 1.10 | 0.031 | 0.39 | 8557 | 4998 |
| DGB 6 | 6/11/13 | 44.016969 | -71.322302 | Douglas Brook | 3137 | 65.8 | 1.65 | 4.81 | 0.033 | 0.056 | 0.14 | 3669 | 2704 |
| EMB 6 | 6/11/13 | 44.737083 | -71.439722 | Emerson Brook | 2091 | 26.6 | 1.87 | 4.83 | 0.074 | 0.093 | 0.11 | 2381 | 1502 |
| HBF 6 | 6/11/13 | 43.95305 | -71.72398 | Paradise Brook | 2189 | 30.2 | 1.42 | 8.80 | 0.257 | 0.074 | 0.07 | 2951 | 3096 |
| HOB 6 | 6/11/13 | 43.082111 | -70.796 | Hodgson Brook | 23734 | 106 | 4.60 | 10.24 | 0.982 | 0.041 | 0.28 | 5011 | 38672 |
| IDM 6 | 6/11/13 | 42.6597 | -70.8942 | Ipswich Dam | 27600 | 440 | 2.66 | 5.43 | 1.136 | 0.071 | 0.38 | 11982 | 49521 |
| IRD 6 | 6/11/13 | 44.411635 | -71.496001 | Israel River | 4225 | 140 | 1.53 | 2.56 | 0.182 | 0.014 | 0.16 | 4091 | 5194 |
| IRU 6 | 6/11/13 | 44.361415 | -71.397224 | Israel River | 2448 | 63.3 | 1.21 | 1.37 | 0.134 | 0.031 | 0.05 | 2370 | 2157 |
| JOB 6 | 6/11/13 | 44.758194 | -71.427028 | Johnson Brook | 2203 | 117 | 2.68 | 3.56 | 0.077 | 0.050 | 0.45 | 3403 | 1676 |
| LND 6 | 6/11/13 | 44.04173 | -71.664205 | Pemigewasset (East) | 3505 | 36.0 | 1.61 | 6.62 | 0.178 | 0.059 | 0.10 | 2585 | 3333 |
| LNU 6 | 6/11/13 | 44.063814 | -71.590948 | Pemigewasset (East) | 2491 | 44.0 | 2.02 | 36.82 | 0.242 | 0.225 | 1.94 | 2887 | 3023 |
| MMR 6 | 6/11/13 | 43.454417 | -71.227917 | Merrymeeting River | 7835 | 197 | 1.61 | 3.81 | 0.855 | 0.021 | 0.219 | 4923 | 9548 |
| MOD 6 | 6/11/13 | 44.373481 | -71.296647 | Moose River | 5057 | 34.3 | 2.55 | 5.99 | 0.348 | 0.040 | 0.133 | 2331 | 5657 |
| MOU 6 | 6/11/13 | 44.373392 | -71.298639 | Moose River | 8168 | 107 | 1.48 | 1.79 | bdl | 0.021 | 0.051 | 2755 | 11214 |
| MRC 6 | 6/11/13 | 43.940361 | -71.511194 | Mad River | 5046 | 83.0 | 1.36 | 7.04 | 0.405 | 0.070 | 0.259 | 2836 | 6515 |
| MRL 6 | 6/11/13 | 43.969111 | -71.50725 | Mad River | 2919 | 47.6 | 1.39 | 6.01 | 0.228 | 0.048 | 0.059 | 3110 | 3024 |
| NEA 6 | 6/11/13 | 43.176972 | -71.831222 | Contoocook | 6927 | 112 | 1.51 | 3.78 | 0.232 | 0.037 | 0.184 | 6064 | 9005 |
| NEB 6 | 6/11/13 | 43.178361 | -71.822028 | Contoocook | 10180 | 219 | 1.73 | 4.88 | 0.340 | 0.068 | 0.506 | 5801 | 13796 |
| NED 6 | 6/11/13 | 43.17375 | -71.816833 | Contoocook | 7760 | 219 | 1.64 | 3.57 | 0.155 | 0.025 | 0.346 | 6332 | 9425 |
| NEU 6 | 6/11/13 | 43.173472 | -71.817472 | Contoocook | 8053 | 322 | 6.43 | 4.25 | 0.321 | 0.037 | 0.450 | 6036 | 10121 |
| NSB 6 | 6/11/13 | 42.740444 | -71.471861 | Salmon Brook | 23088 | 227 | 2.17 | 3.63 | 1.95 | 0.033 | 0.144 | 7368 | 43574 |
| NWD 6 | 6/11/13 | 43.081194 | -70.797917 | Newfields Ditch | 23001 | 235 | 3.07 | 9.59 | 1.10 | 0.051 | 0.684 | 4951 | 39619 |
| OBG 6 | 6/11/13 | 42.917456 | -71.916053 | Otter Brook | 9022 | 226 | 1.79 | 4.50 | 0.360 | 0.035 | 0.268 | 12304 | 12603 |
| OSS 6 | 6/11/13 | 43.79239 | -70.99248 | Ossipee River | 5311 | 57.0 | 1.20 | 2.12 | 0.281 | 0.025 | 0.108 | 2808 | 5995 |
| PBB 6 | 6/11/13 | 43.994875 | -71.352244 | Pine Bend Brook | bdl | 10.5 | 0.198 | 0.46 | 0.049 | 0.006 | 0.038 | 7928 | 3117 |
| PBI 6 | 6/11/13 | 42.978833 | -71.473139 | Piscataquog | 11724 | 201 | 2.27 | 5.77 | 0.688 | 0.034 | 0.207 | 6174 | 16395 |
| PDM 6 | 6/11/13 | 42.7528 | -70.9461 | Parker Dam | 22074 | 460 | 2.17 | 4.32 | 1.532 | 0.059 | 0.335 | 9926 | 37727 |
| PIN 6 | 6/11/13 | 43.76196 | -71.13488 | Pine River | 6134 | 121 | 1.86 | 3.52 | 0.167 | 0.032 | 0.112 | 6409 | 7831 |
| PRF 6 | 6/11/13 | 43.437833 | -71.652333 | Pemigewasset | 6729 | 115 | 1.47 | 3.25 | 0.191 | 0.032 | 0.186 | 2776 | 8396 |
| PRG 6 | 6/11/13 | 43.017306 | -71.601167 | Piscataquog (Main) | 9551 | 245 | 2.51 | 4.09 | 0.768 | 0.036 | 0.348 | 6087 | 14416 |
| PRP 6 | 6/11/13 | 43.760225 | -71.686503 | Pemigewasset | 6552 | 121 | 1.50 | 4.31 | 0.308 | 0.040 | 0.152 | 2714 | 8301 |
| PRW 6 | 6/11/13 | 44.037967 | -71.686885 | Pemigewasset | 5970 | 118 | 1.99 | 5.37 | 0.370 | 0.058 | 0.310 | 4707 | 9278 |
| SBK 6 | 6/11/13 | 42.5236 | -71.185 | Sawmill Brook | 31421 | 331 | 4.39 | 12.03 | 0.924 | 0.071 | 1.190 | 10013 | 57585 |
| SBM 6 | 6/11/13 | 43.170561 | -71.217811 | Saddleback | 4378 | 12.3 | 11.76 | 9.54 | bdl | 0.032 | 0.074 | 2688 | 4199 |
| SCS 6 | 6/11/13 | 44.881867 | -71.072094 | Swift Diamond River | 1859 | 78.2 | 1.36 | 2.72 | 0.234 | 0.034 | 0.110 | 4173 | 2993 |
| SHB 6 | 6/11/13 | 42.954694 | -71.793389 | School House Brook | 3608 | 62.7 | 2.06 | 3.76 | 0.424 | 0.034 | 0.159 | 4985 | 2401 |
| SLB 6 | 6/11/13 | 44.665389 | -71.457417 | Slide Brook | 2538 | 36.1 | 1.22 | 4.32 | 0.280 | 0.062 | 0.080 | 3627 | 1573 |
| SQB 6 | 6/11/13 | 43.703639 | -71.501333 | Unnamed | 9362 | 168 | 1.97 | 2.87 | 0.134 | 0.033 | 0.276 | 5989 | 12602 |
| SQM 6 | 6/11/13 | 43.723806 | -71.559556 | Mill Brook | 6079 | 86.3 | 3.58 | 4.68 | 0.691 | 0.010 | 0.200 | 4242 | 8185 |
| SRM 6 | 6/11/13 | 43.988306 | -71.327444 | Swift River | 3114 | 64.1 | 1.04 | 4.74 | bdl | 0.026 | 0.114 | 3024 | 2762 |
| SRN 6 | 6/11/13 | 43.391472 | -72.186306 | Sugar River | 9648 | 143 | 1.65 | 1.48 | 0.432 | 0.038 | 0.099 | 5428 | 13804 |
| SRS 6 | 6/11/13 | 43.389487 | -72.19456 | Sugar River | 10484 | 133 | 2.01 | 2.52 | 0.364 | 0.033 | 0.276 | 3923 | 14496 |
| SRU 6 | 6/11/13 | 44.023588 | -71.435663 | Swift River | 6190 | 62.2 | 1.91 | 9.43 | 0.215 | 0.093 | 0.356 | 6440 | 7868 |
| SWP 6 | 6/11/13 | 43.756861 | -71.68725 | Storm Drain | 52760 | 81.8 | 5.96 | 18.36 | 0.480 | 0.279 | 0.219 | 3525 | 93326 |
| WBG 6 | 6/11/13 | 43.0165 | -71.60125 | Piscataquog (Main) | 9713 | 504 | 1.24 | 2.59 | 3.39 | 0.038 | 0.604 | 5924 | 13409 |
| WHB 6 | 6/11/13 | 43.122344 | -71.004961 | Wednesday Hill Brook | 17636 | 2305 | 3.93 | 11.42 | 18.14 | 0.082 | 2.770 | 9733 | 22386 |
| WIN 6 | 6/11/13 | 43.440067 | -71.651669 | Winnipesaukee | 11544 | 98.1 | 2.24 | 4.75 | 0.182 | 0.023 | 0.285 | 2787 | 18986 |
| ARD 7 | 7/16/13 | 44.460618 | -71.186188 | Androscoggin | 2556 | 149 | 2.75 | 4.84 | 0.746 | 0.031 | 0.097 | 4965 | 2343 |
| ARU 7 | 7/16/13 | 44.503668 | -71.156587 | Androscoggin | 3105 | 154 | 1.92 | 1.88 | 0.436 | 0.027 | 0.141 | 5738 | 3761 |
| BAT 7 | 7/16/13 | 43.070944 | -70.783111 | Borthwick Ave | bdl | 347 | 5.34 | 0.81 | 0.691 | 0.018 | bdl | 10820 | 349001 |
| BBD 7 | 7/16/13 | 42.923317 | -72.2726 | Beaver Brook | 32822 | 1049 | 3.08 | 5.38 | 0.785 | 0.036 | 1.36 | 6828 | 57000 |
| BBK 7 | 7/16/13 | 42.907736 | -71.922104 | Bogle Brook | 4732 | 409 | 1.95 | 5.13 | 0.694 | 0.019 | 0.499 | 4203 | 5396 |
| BBU 7 | 7/16/13 | 42.957194 | -72.266417 | Beaver Brook | 12248 | 890 | 1.33 | 2.47 | 0.465 | 0.018 | 0.464 | 6142 | 18956 |
| BBW 7 | 7/16/13 | 42.930722 | -72.271444 | Beaver Brook | 24458 | 1169 | 26.62 | 3.47 | 0.992 | 0.036 | 1.00 | 5465 | 42230 |
| BDC 5 | 5/14/13 | 43.094303 | -70.987578 | Burley Demerrit | 9135 | 238 | 2.60 | 7.05 | 2.04 | 0.024 | 0.113 | 11896 | 14449 |
| BDC 7 | 7/16/13 | 43.094303 | -70.987578 | Burley Demerrit | 10519 | 517 | 3.30 | 26.96 | 4.06 | 0.092 | 0.253 | 14210 | 16944 |
| BDD 7 | 7/16/13 | 43.549972 | -72.26775 | Blood Brook | 10433 | 77.4 | 1.12 | bdl | 0.473 | 0.006 | bdl | 2073 | 16721 |
| BDU 7 | 7/16/13 | 43.546528 | -72.270667 | Blood Brook | 10840 | 107 | 1.07 | 1.53 | 0.461 | 0.015 | bdl | 2096 | 16501 |
| BLD 7 | 7/16/13 | 45.050556 | -71.385694 | Back Lake Brook | 7061 | 400 | 1.78 | bdl | 1.02 | 0.009 | 0.139 | 5352 | 10378 |
| BLU 7 | 7/16/13 | 45.052389 | -71.383472 | Back Lake Brook | 4499 | 473 | 0.730 | bdl | 0.559 | 0.018 | 0.169 | 4957 | 6019 |
| BRD 7 | 7/16/13 | 43.1795 | -70.887 | Bellamy River | 14853 | 977 | 0.832 | bdl | 2.98 | 0.009 | 0.440 | 2578 | 24012 |
| BRU 7 | 7/16/13 | 43.178861 | -70.890778 | Bellamy River | 15130 | 1019 | 1.10 | 6.90 | 2.33 | 0.016 | 0.443 | 9759 | 23626 |
| BSW 7 | 7/16/13 | 42.924361 | -72.270389 | Baker St. Storm Water | bdl | 138 | 5.41 | 7.21 | 0.03 | 0.085 | 0.144 | 1248 | 304504 |
| BWS 7 | 7/16/13 | 44.060472 | -71.292917 | Louisville Brook | 5668 | 35.4 | 0.537 | 2.35 | 0.20 | 0.025 | 0.006 | 1973 | 1468 |
| CBT 7 | 7/16/13 | 43.771667 | -71.72989 | Clay Brook | 5090 | 345 | 1.60 | 2.17 | 0.25 | 0.019 | 0.199 | 5553 | 6225 |
| CBU 7 | 7/16/13 | 43.7454 | -71.719 | Clay Brook | 3561 | 337 | 1.34 | 1.37 | 0.33 | 0.030 | 0.187 | 4092 | 4403 |
| CCK 7 | 7/16/13 | 42.7692 | -70.9165 | Cart Creek | 48108 | 1868 | 7.24 | 2.63 | 5.59 | 0.057 | 1.01 | 11283 | 84145 |
| CRD 7 | 7/16/13 | 43.800917 | -71.437472 | Creamery Brook | 21934 | 202 | 1.92 | 3.17 | 0.358 | 0.013 | 0.135 | 4147 | 39264 |
| CRU 7 | 7/16/13 | 43.803889 | -71.442278 | Creamery Brook | 16447 | 80.1 | 1.31 | bdl | 0.245 | 0.004 | bdl | 3380 | 25733 |
| CSP 7 | 7/16/13 | 42.5495 | -71.0909 | Cedar Swamp | 29260 | 2273 | 2.74 | 4.23 | 3.32 | 0.061 | 1.61 | 45470 | 61601 |
| CTC 7 | 7/16/13 | 45.022083 | -71.46275 | Connecticut | 2586 | 56.3 | 0.826 | bdl | 0.246 | 0.004 | bdl | 4708 | 2385 |
| CTP 7 | 7/16/13 | 45.061611 | -71.298806 | Connecticut | 2009 | 59.3 | 1.70 | 1.39 | 0.291 | 0.016 | 0.075 | 4567 | 2031 |
| DCF 7 | 7/16/13 | 43.13483 | -71.18219 | Dowst-Cate | 5048 | 667 | 0.738 | 0.82 | 1.49 | 0.009 | 0.232 | 8762 | 7777 |
| EMB 7 | 7/16/13 | 44.737083 | -71.439722 | Emerson Brook | 1813 | 23.6 | 0.564 | 0.97 | 0.057 | 0.042 | 0.077 | 1105 | 1352 |
| EXT 7 | 7/16/13 | 42.982847 | -70.949131 | Exeter River | 17358 | 433 | 1.54 | 15.60 | 1.85 | 0.019 | 0.223 | 10158 | 27703 |
| HBF 7 | 7/16/13 | 43.95305 | -71.72398 | Paradise Brook | 2434 | 22.2 | 3.78 | 9.82 | 0.155 | 0.041 | 0.096 | 2490 | 1591 |
| HOB 7 | 7/16/13 | 43.082111 | -70.796 | Hodgson Brook | bdl | 439 | 4.65 | 0.46 | 2.91 | 0.039 | 0.153 | 3042 | 310018 |
| IDM 7 | 7/16/13 | 42.6597 | -70.8942 | Ipswich Dam | 35672 | 732 | 2.56 | 5.95 | 2.08 | 0.094 | 0.256 | 12244 | 66221 |
| IRD 7 | 7/16/13 | 44.411635 | -71.496001 | Israel River | 4157 | 254 | 0.980 | 0.15 | 0.122 | bdl | 0.093 | 4357 | 6057 |
| IRU 7 | 7/16/13 | 44.361415 | -71.397224 | Israel River | 2507 | 45.1 | 0.535 | bdl | 0.176 | 0.015 | 0.069 | 2444 | 4011 |
| JOB 7 | 7/16/13 | 44.758194 | -71.427028 | Johnson Brook | 2164 | 43.6 | 1.22 | 1.84 | 0.019 | 0.027 | 0.070 | 1561 | 3044 |
| LND 7 | 7/16/13 | 44.04173 | -71.664205 | Pemigewasset (East) | 3404 | 42.6 | 1.39 | 5.23 | 0.125 | 0.056 | 0.107 | 2035 | 3125 |
| LNU 7 | 7/16/13 | 44.063814 | -71.590948 | Pemigewasset (East) | 2390 | 43.1 | 0.802 | 11.15 | 0.086 | 0.050 | 0.132 | 2294 | 1446 |
| MMR 7 | 7/16/13 | 43.454417 | -71.227917 | Merrymeeting River | 7481 | 375 | 0.776 | 4.69 | 1.45 | 0.008 | 0.136 | 7013 | 9829 |
| MOD 7 | 7/16/13 | 44.373481 | -71.296647 | Moose River | 5601 | 139 | 2.78 | 4.57 | 0.447 | 0.037 | 0.202 | 2445 | 7493 |
| MOU 7 | 7/16/13 | 44.373392 | -71.298639 | Moose River | 9878 | 214 | 1.60 | 3.41 | 0.194 | 0.012 | 0.139 | 2337 | 13852 |
| MRC 7 | 7/16/13 | 43.940361 | -71.511194 | Mad River | 8004 | 61.9 | 1.58 | 6.06 | 0.128 | 0.072 | 0.100 | 1865 | 9690 |
| MRL 7 | 7/16/13 | 43.969111 | -71.50725 | Mad River | 2891 | 31.0 | 0.616 | 2.43 | 0.331 | 0.058 | bdl | 1865 | 1361 |
| NEA 7 | 7/16/13 | 43.176972 | -71.831222 | Contoocook | 8866 | 522 | 0.877 | 2.44 | 0.496 | 0.013 | 0.455 | 5079 | 12864 |
| NEB 7 | 7/16/13 | 43.178361 | -71.822028 | Contoocook | 10505 | 502 | 1.99 | 3.02 | 0.614 | 0.031 | 0.471 | 5841 | 13640 |
| NED 7 | 7/16/13 | 43.17375 | -71.816833 | Contoocook | 11281 | 535 | 0.669 | 0.991 | 0.737 | 0.011 | 0.495 | 5749 | 15192 |
| NEU 7 | 7/16/13 | 43.173472 | -71.817472 | Contoocook | 14993 | 453 | 1.19 | 4.15 | 0.904 | 0.024 | 0.378 | 5632 | 22130 |
| NSB 7 | 7/16/13 | 42.740444 | -71.471861 | Salmon Brook | 29032 | 191 | 4.70 | bdl | 1.84 | bdl | 0.030 | 5427 | 60004 |
| NWD 7 | 7/16/13 | 43.081194 | -70.797917 | Newfields Ditch | 105851 | 482 | 4.40 | 4.97 | 3.69 | 0.047 | 0.258 | 2593 | 210889 |
| OBG 7 | 7/16/13 | 42.917456 | -71.916053 | Otter Brook | 11529 | 254 | 0.754 | 1.55 | 0.935 | 0.016 | 0.049 | 8264 | 17902 |
| OSS 7 | 7/16/13 | 43.79239 | -70.99248 | Ossipee River | 5317 | 89.0 | 1.07 | 3.79 | 0.342 | 0.010 | 0.037 | 3322 | 7577 |
| PBB 7 | 7/16/13 | 43.994875 | -71.352244 | Pine Bend Brook | 2610 | 36.8 | 20.53 | 0.922 | 0.049 | bdl | 0.023 | 2031 | 1666 |
| PBD 7 | 7/16/13 | 42.969694 | -71.697861 | Piscataquog | 12257 | 494 | 0.882 | bdl | 1.49 | 0.020 | 0.242 | 5580 | 15593 |
| PBI 7 | 7/16/13 | 42.978833 | -71.473139 | Piscataquog | 13278 | 442 | 1.89 | 4.61 | 1.87 | 0.011 | 0.357 | 5940 | 20483 |
| PBU 7 | 7/16/13 | 42.979889 | -71.689139 | Piscataquog | 10279 | 612 | 2.12 | 2.69 | 1.64 | 0.032 | 0.347 | 5877 | 11850 |
| PDM 7 | 7/16/13 | 42.7528 | -70.9461 | Parker Dam | 22882 | 663 | 1.00 | bdl | 2.25 | 0.024 | 0.157 | 10236 | 37524 |
| PIN 7 | 7/16/13 | 43.76196 | -71.13488 | Pine River | 7269 | 387 | 1.66 | 2.47 | 0.405 | 0.027 | 0.272 | 5321 | 9125 |
| PRF 7 | 7/16/13 | 43.437833 | -71.652333 | Pemigewasset | 5561 | 149 | 0.96 | 6.95 | 0.228 | 0.049 | 0.144 | 4725 | 7155 |
| PRG 7 | 7/16/13 | 43.017306 | -71.601167 | Piscataquog (Main) | 11232 | 479 | 1.76 | 1.94 | 1.27 | 0.025 | 0.291 | 5079 | 14357 |
| PRP 7 | 7/16/13 | 43.760225 | -71.686503 | Pemigewasset | 6524 | 164 | 1.91 | 2.72 | 0.252 | 0.012 | 0.118 | 1323 | 5991 |
| PRW 7 | 7/16/13 | 44.037967 | -71.686885 | Pemigewasset | 8145 | 64.2 | 1.40 | 2.51 | 0.319 | 0.0363 | 0.080 | 1939 | 10445 |
| PWU 7 | 7/16/13 | 43.109028 | -71.762039 | Piscataquog (North) | 6494 | 161 | 0.811 | 3.07 | 0.952 | 0.0135 | 0.047 | 5382 | 8691 |
| SBK 7 | 7/16/13 | 42.5236 | -71.185 | Sawmill Brook | 131024 | 570 | 5.32 | 9.51 | 0.926 | 0.0894 | 0.660 | 4701 | 267598 |
| SBM 7 | 7/16/13 | 43.170561 | -71.217811 | Saddleback | 4463 | 24.8 | 0.94 | 614.22 | 0.286 | 0.0301 | 0.003 | 971 | 5689 |
| SCD 7 | 7/16/13 | 44.88275 | -71.071956 | Dead Diamond River | 2082 | 175 | 0.98 | bdl | 0.912 | 0.0232 | 0.014 | 3201 | 3023 |
| SCS 7 | 7/16/13 | 44.881867 | -71.072094 | Swift Diamond River | 2048 | 120 | 1.47 | 1.92 | 0.055 | 0.0300 | 0.112 | 2957 | 1320 |
| SHB 7 | 7/16/13 | 42.954694 | -71.793389 | School House Brook | 3629 | 42.3 | 0.739 | bdl | 0.536 | 0.0202 | 0.032 | 1628 | 2827 |
| SLB 7 | 7/16/13 | 44.665389 | -71.457417 | Slide Brook | 2473 | 47.2 | 1.29 | 2.29 | 0.122 | 0.0097 | 0.069 | 2189 | 3064 |
| SQB 7 | 7/16/13 | 43.703639 | -71.501333 | Unnamed | 12464 | 294 | 1.08 | 21.52 | 0.568 | 0.0297 | 0.182 | 5769 | 19520 |
| SQM 7 | 7/16/13 | 43.723806 | -71.559556 | Mill Brook | 7809 | 71.5 | 4.78 | bdl | 0.163 | 0.0113 | 0.058 | 4259 | 11559 |
| SQR 7 | 7/16/13 | 42.981383 | -70.944344 | Squamscott River | 18723 | 816 | 2.58 | 4.37 | 3.16 | 0.128 | 0.946 | 10386 | 31814 |
| SRM 7 | 7/16/13 | 43.988306 | -71.327444 | Swift River | 3388 | 88.6 | 32.59 | 3.53 | 0.189 | 0.0348 | 0.125 | 2808 | 4467 |
| SRN 7 | 7/16/13 | 43.391472 | -72.186306 | Sugar River | 12102 | 480 | 1.13 | 2.81 | 0.662 | 0.0241 | 0.155 | 5364 | 19259 |
| SRS 7 | 7/16/13 | 43.389487 | -72.19456 | Sugar River | 11658 | 98.5 | 1.19 | 9.60 | 0.306 | 0.0187 | 0.064 | 3331 | 18344 |
| SRU 7 | 7/16/13 | 44.023588 | -71.435663 | Swift River | 5414 | 44.8 | 0.668 | 10.33 | 0.214 | 0.0637 | 0.042 | 3314 | 6659 |
| SWP 7 | 7/16/13 | 43.756861 | -71.68725 | Storm Drain | bdl | 311 | 9.04 | 100.15 | 0.268 | 0.207 | 1.310 | 1606 | 418745 |
| WBG 7 | 7/16/13 | 43.0165 | -71.60125 | Piscataquog (Main) | 13184 | 730 | 1.02 | 1.24 | 6.06 | bdl | 0.363 | 5346 | 19212 |
| WHB 7 | 7/16/13 | 43.122344 | -71.004961 | Wednesday Hill Brook | 32602 | 186 | 1.35 | bdl | 7.02 | 0.0527 | 0.027 | 4411 | 50863 |
| WIN 7 | 7/16/13 | 43.440067 | -71.651669 | Winnipesaukee | 10674 | 81.2 | 1.18 | 296.71 | 0.266 | 3.54 | 0.826 | 2695 | 15975 |

**Table B. Land use and landscape site-specific information.** Site characteristics across northern New England; sites marked with (*) include those samples removed from subsequent statistical analysis.

| Site ID | Date | Latitude | Longitude | River | Area, sq/km | Development, % | Agriculture, % | Forest, % | Open Water, % | Wetland, % | Bare, % | Basin Elevation, m |
| --- | --- | --- | --- | --- | --- | --- | --- | --- | --- | --- | --- | --- |
| ARD 5 | 5/14/13 | 44.460618 | -71.186188 | Androscoggin | 3504 | 1.32 | 0.11 | 85.99 | 6.27 | 5.78 | 0.50 | 576.70 |
| ARU 5 | 5/14/13 | 44.503668 | -71.156587 | Androscoggin | 3438 | 1.11 | 0.11 | 86.09 | 6.37 | 5.83 | 0.47 | 578.80 |
| BAT 5 | 5/14/13 | 43.070944 | -70.783111 | Borthwick Ave | 10.59 | 81.69 | 1.01 | 7.61 | 0.14 | 9.55 | 0.00 | 14.90 |
| BBD 5 | 5/14/13 | 42.923317 | -72.2726 | Beaver Brook | 24.14 | 18.10 | 1.89 | 75.18 | 0.09 | 4.64 | 0.09 | 279.70 |
| BBK 5 | 5/14/13 | 42.907736 | -71.922104 | Bogle Brook | 10.72 | 3.43 | 3.79 | 88.55 | 0.39 | 3.85 | 0.00 | 364.80 |
| BBU 5 | 5/14/13 | 42.957194 | -72.266417 | Beaver Brook | 16.99 | 5.85 | 2.17 | 85.84 | 0.06 | 5.95 | 0.13 | 311.00 |
| BBW 5 | 5/14/13 | 42.930722 | -72.271444 | Beaver Brook | 21.89 | 14.67 | 2.06 | 78.09 | 0.05 | 5.04 | 0.10 | 288.00 |
| BDU 5 | 5/14/13 | 43.546528 | -72.270667 | Blood Brook | 31.49 | 3.35 | 6.67 | 86.46 | 0.07 | 3.42 | 0.03 | 445.30 |
| BLD 5 | 5/14/13 | 45.050556 | -71.385694 | Back Lake Brook | 6.35 | 6.95 | 0.00 | 73.72 | 11.31 | 7.92 | 0.10 | 484.50 |
| BLU 5 | 5/14/13 | 45.052389 | -71.383472 | Back Lake Brook | 6.19 | 6.39 | 0.00 | 74.12 | 11.44 | 7.98 | 0.10 | 485.40 |
| BRD 5 | 5/14/13 | 43.1795 | -70.887 | Bellamy River | 67.60 | 13.11 | 6.90 | 55.02 | 4.05 | 18.19 | 2.72 | 65.10 |
| BRU 5 | 5/14/13 | 43.178861 | -70.890778 | Bellamy River | 66.98 | 25.28 | 15.24 | 38.89 | 0.04 | 14.13 | 6.41 | 65.50 |
| BWS 5 | 5/14/13 | 42.924361 | -72.270389 | Louisville Brook | 7.51 | 0.92 | 0.00 | 99.02 | 0.00 | 0.06 | 0.00 | 515.00 |
| CBT 5 | 5/14/13 | 43.771667 | -71.72989 | Clay Brook | 11.14 | 1.84 | 4.63 | 86.36 | 0.00 | 6.97 | 0.19 | 316.80 |
| CBU 5 | 5/14/13 | 43.7454 | -71.719 | Clay Brook | 7.49 | 1.29 | 5.17 | 89.28 | 0.00 | 4.26 | 0.00 | 359.20 |
| CCK 5 | 5/14/13 | 42.7692 | -70.9165 | Cart Creek | 1.66 | 4.10 | 5.23 | 46.76 | 0.00 | 43.91 | 0.00 | 16.20 |
| CRD 5 | 5/14/13 | 43.800917 | -71.437472 | Creamery Brook | 2.15 | 11.61 | 6.97 | 78.90 | 0.00 | 2.53 | 0.00 | 246.10 |
| CRU 5 | 5/14/13 | 43.803889 | -71.442278 | Creamery Brook | 1.68 | 8.43 | 0.00 | 87.39 | 0.00 | 4.19 | 0.00 | 258.60 |
| CSP 5 | 5/14/13 | 42.5495 | -71.0909 | Cedar Swamp | 3.16 | 24.66 | 1.69 | 24.66 | 0.00 | 43.16 | 0.72 | 28.50 |
| DCF 5 | 5/14/13 | 43.13483 | -71.18219 | Dowst-Cate | 7.17 | 5.35 | 3.40 | 77.77 | 1.72 | 11.64 | 0.13 | 152.80 |
| DGB 5 | 5/14/13 | 44.016969 | -71.322302 | Douglas Brook | 1.24 | 0.22 | 0.00 | 99.78 | 0.00 | 0.00 | 0.00 | 682.30 |
| EMB 5 | 5/14/13 | 44.737083 | -71.439722 | Emerson Brook | 1.09 | 0.00 | 0.00 | 100.00 | 0.00 | 0.00 | 0.00 | 829.00 |
| HOB 5 | 5/14/13 | 43.082111 | -70.796 | Hodgson Brook | 5.23 | 91.18 | 0.00 | 3.30 | 0.00 | 5.50 | 0.00 | 20.60 |
| IDM 5 | 5/14/13 | 42.6597 | -70.8942 | Ipswich Dam | 317.17 | 37.10 | 2.13 | 35.33 | 2.21 | 22.86 | 0.36 | 32.90 |
| IRD 5 | 5/14/13 | 44.411635 | -71.496001 | Israel River | 186.38 | 2.68 | 2.05 | 89.96 | 0.06 | 4.64 | 0.00 | 625.10 |
| IRU 5 | 5/14/13 | 44.361415 | -71.397224 | Israel River | 53.38 | 1.80 | 0.01 | 95.32 | 0.00 | 0.80 | 2.08 | 742.30 |
| JOB 5 | 5/14/13 | 44.758194 | -71.427028 | Johnson Brook | 1.74 | 0.00 | 0.00 | 100.00 | 0.00 | 0.00 | 0.00 | 822.70 |
| MOD 5 | 5/14/13 | 44.373481 | -71.296647 | Moose River | 21.50 | 3.23 | 0.00 | 93.49 | 0.00 | 1.30 | 1.99 | 810.70 |
| MOU 5 | 5/14/13 | 44.373392 | -71.298639 | Moose River | 15.00 | 4.63 | 0.12 | 93.42 | 0.00 | 1.83 | 0.00 | 589.20 |
| MRC 5 | 5/14/13 | 43.940361 | -71.511194 | Mad River | 65.16 | 3.15 | 0.00 | 96.45 | 0.06 | 0.32 | 0.03 | 755.70 |
| MRL 5 | 5/14/13 | 43.969111 | -71.50725 | Mad River | 15.51 | 0.52 | 0.00 | 99.00 | 0.16 | 0.32 | 0.00 | 807.80 |
| NEA 5 | 5/14/13 | 43.176972 | -71.831222 | Contoocook | 974.28 | 6.29 | 3.94 | 77.60 | 3.21 | 8.75 | 0.22 | 342.90 |
| NEB 5 | 5/14/13 | 43.178361 | -71.822028 | Contoocook | 976.38 | 6.33 | 3.94 | 77.57 | 3.21 | 8.74 | 0.22 | 342.60 |
| NED 5 | 5/14/13 | 43.17375 | -71.816833 | Contoocook | 976.28 | 6.37 | 3.94 | 77.54 | 3.21 | 8.74 | 0.21 | 342.60 |
| NEU 5 | 5/14/13 | 43.173472 | -71.817472 | Contoocook | 975.89 | 6.35 | 3.94 | 77.55 | 3.21 | 8.74 | 0.21 | 342.60 |
| NSB 5 | 5/14/13 | 42.740444 | -71.471861 | Salmon Brook | 68.25 | 21.85 | 7.48 | 51.09 | 4.02 | 15.23 | 0.33 | 73.60 |
| NWD 5 | 5/14/13 | 43.081194 | -70.797917 | Newfields Ditch | 3.60 | 95.26 | 0.00 | 0.89 | 0.00 | 3.85 | 0.00 | 22.20 |
| OBG 5 | 5/14/13 | 42.917456 | -71.916053 | Otter Brook | 30.04 | 5.03 | 4.65 | 71.99 | 3.35 | 14.64 | 0.34 | 272.90 |
| OSS 5 | 5/14/13 | 43.79239 | -70.99248 | Ossipee River | 904.48 | 4.26 | 1.47 | 83.24 | 3.59 | 6.73 | 0.70 | 293.40 |
| PBB 5 | 5/14/13 | 43.994875 | -71.352244 | Pine Bend Brook | 2.64 | 0.00 | 0.00 | 87.60 | 0.48 | 11.93 | 0.00 | 469.20 |
| PBD 5 | 5/14/13 | 42.969694 | -71.697861 | Piscataquog | 140.97 | 5.83 | 4.68 | 79.95 | 0.87 | 8.21 | 0.45 | 258.90 |
| PBI 5 | 5/14/13 | 42.978833 | -71.473139 | Piscataquog | 403.63 | 9.11 | 5.65 | 75.70 | 1.26 | 7.76 | 0.52 | 205.20 |
| PBU 5 | 5/14/13 | 42.979889 | -71.689139 | Piscataquog | 143.12 | 6.07 | 4.77 | 79.71 | 0.86 | 8.13 | 0.45 | 257.60 |
| PIN 5 | 5/14/13 | 43.76196 | -71.13488 | Pine River | 227.30 | 4.65 | 1.48 | 80.05 | 2.63 | 9.99 | 1.20 | 234.40 |
| PRF 5 | 5/14/13 | 43.437833 | -71.652333 | Pemigewasset | 2646.46 | 3.35 | 1.91 | 89.30 | 2.62 | 2.59 | 0.22 | 447.60 |
| PRG 5 | 5/14/13 | 43.017306 | -71.601167 | Piscataquog (Main) | 332.12 | 6.40 | 5.50 | 78.27 | 1.00 | 8.27 | 0.57 | 218.90 |
| PWU 5 | 5/14/13 | 43.109028 | -71.762039 | Piscataquog (North) | 79.12 | 5.21 | 5.38 | 77.82 | 4.27 | 7.21 | 0.10 | 284.10 |
| SBK 5* | 5/14/13 | 42.5236 | -71.185 | Sawmill Brook | 4.09 | 87.85 | 0.00 | 8.21 | 0.00 | 3.01 | 0.00 | 47.90 |
| SBM 5 | 5/14/13 | 43.170561 | -71.217811 | Saddleback | 0.49 | 6.32 | 0.00 | 93.68 | 0.00 | 0.00 | 0.00 | 256.50 |
| SCD 5 | 5/14/13 | 44.88275 | -71.071956 | Dead Diamond River | 217.17 | 0.27 | 0.00 | 95.37 | 0.06 | 4.27 | 0.03 | 632.50 |
| SCS 5* | 5/14/13 | 44.881867 | -71.072094 | Swift Diamond River | 178.68 | 0.43 | 0.00 | 95.97 | 0.67 | 2.88 | 0.05 | 664.60 |
| SHB 5 | 5/14/13 | 42.954694 | -71.793389 | School House Brook | 1.50 | 4.70 | 0.48 | 94.52 | 0.00 | 0.30 | 0.00 | 333.20 |
| SLB 5 | 5/14/13 | 44.665389 | -71.457417 | Slide Brook | 1.37 | 0.00 | 0.00 | 98.40 | 0.00 | 0.00 | 1.60 | 665.50 |
| SQB 5 | 5/14/13 | 43.703639 | -71.501333 | Unnamed | 3.13 | 3.03 | 2.98 | 91.33 | 0.20 | 2.46 | 0.00 | 233.10 |
| SQM 5 | 5/14/13 | 43.723806 | -71.559556 | Mill Brook | 12.15 | 1.64 | 2.48 | 74.70 | 10.27 | 10.91 | 0.00 | 210.40 |
| SRM 5 | 5/14/13 | 43.988306 | -71.327444 | Swift River | 130.87 | 1.06 | 0.00 | 95.95 | 0.10 | 2.84 | 0.05 | 617.30 |
| SRN 5 | 5/14/13 | 43.391472 | -72.186306 | Sugar River | 210.98 | 5.95 | 3.12 | 81.62 | 2.05 | 6.64 | 0.62 | 401.00 |
| SRU 5* | 5/14/13 | 44.023588 | -71.435663 | Swift River | 12.02 | 3.35 | 0.00 | 96.45 | 0.09 | 0.11 | 0.00 | 781.80 |
| WBG 5 | 5/14/13 | 43.0165 | -71.60125 | Piscataquog (Main) | 5.34 | 8.40 | 2.64 | 84.59 | 0.13 | 4.26 | 0.00 | 193.90 |
| WHB 5 | 5/14/13 | 43.122344 | -71.004961 | Wednesday Hill Brook | 1.27 | 24.47 | 8.96 | 60.30 | 0.00 | 6.28 | 0.00 | 47.90 |
| ARU 6 | 6/11/13 | 44.503668 | -71.156587 | Androscoggin | 3437.53 | 1.11 | 0.11 | 86.09 | 6.37 | 5.83 | 0.47 | 578.80 |
| BAT 6 | 6/11/13 | 43.070944 | -70.783111 | Borthwick Ave | 10.59 | 81.69 | 1.01 | 7.61 | 0.14 | 9.55 | 0.00 | 14.90 |
| BBD 6 | 6/11/13 | 42.923317 | -72.2726 | Beaver Brook | 24.14 | 18.10 | 1.89 | 75.18 | 0.09 | 4.64 | 0.09 | 279.70 |
| BBK 6 | 6/11/13 | 42.907736 | -71.922104 | Bogle Brook | 10.72 | 3.43 | 3.79 | 88.55 | 0.39 | 3.85 | 0.00 | 364.80 |
| BBU 6 | 6/11/13 | 42.957194 | -72.266417 | Beaver Brook | 16.99 | 5.85 | 2.17 | 85.84 | 0.06 | 5.95 | 0.13 | 311.00 |
| BBW 6 | 6/11/13 | 42.930722 | -72.271444 | Beaver Brook | 21.89 | 14.67 | 2.06 | 78.09 | 0.05 | 5.04 | 0.10 | 288.00 |
| BDC 6 | 6/11/13 | 43.094303 | -70.987578 | Burley Demerrit | 0.23 | 5.95 | 41.27 | 39.68 | 0.00 | 13.10 | 0.00 | 34.70 |
| BLD 6 | 6/11/13 | 45.050556 | -71.385694 | Back Lake Brook | 6.35 | 6.95 | 0.00 | 73.72 | 11.31 | 7.92 | 0.10 | 484.50 |
| BLU 6 | 6/11/13 | 45.052389 | -71.383472 | Back Lake Brook | 6.19 | 6.39 | 0.00 | 74.12 | 11.44 | 7.98 | 0.10 | 485.40 |
| BRD 6 | 6/11/13 | 43.1795 | -70.887 | Bellamy River | 67.60 | 13.11 | 6.90 | 55.02 | 4.05 | 18.19 | 2.72 | 65.10 |
| BRU 6 | 6/11/13 | 43.178861 | -70.890778 | Bellamy River | 66.98 | 25.28 | 15.24 | 38.89 | 0.04 | 14.13 | 6.41 | 65.50 |
| BWS 6 | 6/11/13 | 42.924361 | -72.270389 | Louisville Brook | 7.51 | 0.92 | 0.00 | 99.02 | 0.00 | 0.06 | 0.00 | 515.00 |
| CBT 6 | 6/11/13 | 43.771667 | -71.72989 | Clay Brook | 11.14 | 1.84 | 4.63 | 86.36 | 0.00 | 6.97 | 0.19 | 316.80 |
| CBU 6 | 6/11/13 | 43.7454 | -71.719 | Clay Brook | 7.49 | 1.29 | 5.17 | 89.28 | 0.00 | 4.26 | 0.00 | 359.20 |
| CCK 6 | 6/11/13 | 42.7692 | -70.9165 | Cart Creek | 1.66 | 4.10 | 5.23 | 46.76 | 0.00 | 43.91 | 0.00 | 16.20 |
| CRD 6 | 6/11/13 | 43.800917 | -71.437472 | Creamery Brook | 2.15 | 11.61 | 6.97 | 78.90 | 0.00 | 2.53 | 0.00 | 246.10 |
| CRU 6 | 6/11/13 | 43.803889 | -71.442278 | Creamery Brook | 1.68 | 8.43 | 0.00 | 87.39 | 0.00 | 4.19 | 0.00 | 258.60 |
| CSP 6 | 6/11/13 | 42.5495 | -71.0909 | Cedar Swamp | 3.16 | 24.66 | 1.69 | 24.66 | 0.00 | 43.16 | 0.72 | 28.50 |
| DCF 6 | 6/11/13 | 43.13483 | -71.18219 | Dowst-Cate | 7.17 | 5.35 | 3.40 | 77.77 | 1.72 | 11.64 | 0.13 | 152.80 |
| DGB 6 | 6/11/13 | 44.016969 | -71.322302 | Douglas Brook | 1.24 | 0.22 | 0.00 | 99.78 | 0.00 | 0.00 | 0.00 | 682.30 |
| EMB 6 | 6/11/13 | 44.737083 | -71.439722 | Emerson Brook | 1.09 | 0.00 | 0.00 | 100.00 | 0.00 | 0.00 | 0.00 | 829.00 |
| HBF 6 | 6/11/13 | 43.95305 | -71.72398 | Paradise Brook | 0.67 | 0.00 | 0.00 | 100.00 | 0.00 | 0.00 | 0.00 | 622.10 |
| HOB 6 | 6/11/13 | 43.082111 | -70.796 | Hodgson Brook | 5.23 | 91.18 | 0.00 | 3.30 | 0.00 | 5.50 | 0.00 | 20.60 |
| IDM 6 | 6/11/13 | 42.6597 | -70.8942 | Ipswich Dam | 317.17 | 37.10 | 2.13 | 35.33 | 2.21 | 22.86 | 0.36 | 32.90 |
| IRD 6 | 6/11/13 | 44.411635 | -71.496001 | Israel River | 186.38 | 2.68 | 2.05 | 89.96 | 0.06 | 4.64 | 0.00 | 625.10 |
| IRU 6 | 6/11/13 | 44.361415 | -71.397224 | Israel River | 53.38 | 1.80 | 0.01 | 95.32 | 0.00 | 0.80 | 2.08 | 742.30 |
| JOB 6 | 6/11/13 | 44.758194 | -71.427028 | Johnson Brook | 1.74 | 0.00 | 0.00 | 100.00 | 0.00 | 0.00 | 0.00 | 822.70 |
| LND 6 | 6/11/13 | 44.04173 | -71.664205 | Pemigewasset (East) | 297.85 | 0.84 | 0.01 | 98.39 | 0.07 | 0.54 | 0.15 | 798.80 |
| LNU 6 | 6/11/13 | 44.063814 | -71.590948 | Pemigewasset (East) | 215.59 | 0.08 | 0.00 | 99.12 | 0.05 | 0.56 | 0.18 | 840.30 |
| MMR 6 | 6/11/13 | 43.454417 | -71.227917 | Merrymeeting River | 88.66 | 6.31 | 2.47 | 73.41 | 6.45 | 11.20 | 0.16 | 230.20 |
| MOD 6 | 6/11/13 | 44.373481 | -71.296647 | Moose River | 21.50 | 3.23 | 0.00 | 93.49 | 0.00 | 1.30 | 1.99 | 810.70 |
| MOU 6 | 6/11/13 | 44.373392 | -71.298639 | Moose River | 15.00 | 4.63 | 0.12 | 93.42 | 0.00 | 1.83 | 0.00 | 589.20 |
| MRC 6 | 6/11/13 | 43.940361 | -71.511194 | Mad River | 65.16 | 3.15 | 0.00 | 96.45 | 0.06 | 0.32 | 0.03 | 755.70 |
| MRL 6 | 6/11/13 | 43.969111 | -71.50725 | Mad River | 15.51 | 0.52 | 0.00 | 99.00 | 0.16 | 0.32 | 0.00 | 807.80 |
| NEA 6 | 6/11/13 | 43.176972 | -71.831222 | Contoocook | 974.28 | 6.29 | 3.94 | 77.60 | 3.21 | 8.75 | 0.22 | 342.90 |
| NEB 6 | 6/11/13 | 43.178361 | -71.822028 | Contoocook | 976.38 | 6.33 | 3.94 | 77.57 | 3.21 | 8.74 | 0.22 | 342.60 |
| NED 6 | 6/11/13 | 43.17375 | -71.816833 | Contoocook | 976.28 | 6.37 | 3.94 | 77.54 | 3.21 | 8.74 | 0.21 | 342.60 |
| NEU 6 | 6/11/13 | 43.173472 | -71.817472 | Contoocook | 975.89 | 6.35 | 3.94 | 77.55 | 3.21 | 8.74 | 0.21 | 342.60 |
| NSB 6 | 6/11/13 | 42.740444 | -71.471861 | Salmon Brook | 68.25 | 21.85 | 7.48 | 51.09 | 4.02 | 15.23 | 0.33 | 73.60 |
| NWD 6 | 6/11/13 | 43.081194 | -70.797917 | Newfields Ditch | 3.60 | 95.26 | 0.00 | 0.89 | 0.00 | 3.85 | 0.00 | 22.20 |
| OBG 6 | 6/11/13 | 42.917456 | -71.916053 | Otter Brook | 30.04 | 5.03 | 4.65 | 71.99 | 3.35 | 14.64 | 0.34 | 272.90 |
| OSS 6 | 6/11/13 | 43.79239 | -70.99248 | Ossipee River | 904.48 | 4.26 | 1.47 | 83.24 | 3.59 | 6.73 | 0.70 | 293.40 |
| PBB 6 | 6/11/13 | 43.994875 | -71.352244 | Pine Bend Brook | 2.64 | 0.00 | 0.00 | 87.60 | 0.48 | 11.93 | 0.00 | 469.20 |
| PBI 6 | 6/11/13 | 42.978833 | -71.473139 | Piscataquog | 403.63 | 9.11 | 5.65 | 75.70 | 1.26 | 7.76 | 0.52 | 205.20 |
| PDM 6 | 6/11/13 | 42.7528 | -70.9461 | Parker Dam | 54.26 | 18.54 | 6.60 | 49.01 | 1.91 | 23.89 | 0.05 | 36.40 |
| PIN 6 | 6/11/13 | 43.76196 | -71.13488 | Pine River | 227.30 | 4.65 | 1.48 | 80.05 | 2.63 | 9.99 | 1.20 | 234.40 |
| PRF 6 | 6/11/13 | 43.437833 | -71.652333 | Pemigewasset | 2646.46 | 3.35 | 1.91 | 89.30 | 2.62 | 2.59 | 0.22 | 447.60 |
| PRG 6 | 6/11/13 | 43.017306 | -71.601167 | Piscataquog (Main) | 332.12 | 6.40 | 5.50 | 78.27 | 1.00 | 8.27 | 0.57 | 218.90 |
| PRP 6 | 6/11/13 | 43.760225 | -71.686503 | Pemigewasset | 1601.99 | 3.12 | 1.25 | 93.06 | 0.42 | 1.88 | 0.27 | 543.70 |
| PRW 6 | 6/11/13 | 44.037967 | -71.686885 | Pemigewasset | 86.66 | 4.49 | 0.02 | 93.25 | 0.22 | 1.36 | 0.66 | 729.80 |
| SBK 6 | 6/11/13 | 42.5236 | -71.185 | Sawmill Brook | 4.09 | 87.85 | 0.00 | 8.21 | 0.00 | 3.01 | 0.00 | 47.90 |
| SBM 6 | 6/11/13 | 43.170561 | -71.217811 | Saddleback | 0.49 | 6.32 | 0.00 | 93.68 | 0.00 | 0.00 | 0.00 | 256.50 |
| SCS 6 | 6/11/13 | 44.881867 | -71.072094 | Swift Diamond River | 178.68 | 0.43 | 0.00 | 95.97 | 0.67 | 2.88 | 0.05 | 664.60 |
| SHB 6 | 6/11/13 | 42.954694 | -71.793389 | School House Brook | 1.50 | 4.70 | 0.48 | 94.52 | 0.00 | 0.30 | 0.00 | 333.20 |
| SLB 6 | 6/11/13 | 44.665389 | -71.457417 | Slide Brook | 1.37 | 0.00 | 0.00 | 98.40 | 0.00 | 0.00 | 1.60 | 665.50 |
| SQB 6 | 6/11/13 | 43.703639 | -71.501333 | Unnamed | 3.13 | 3.03 | 2.98 | 91.33 | 0.20 | 2.46 | 0.00 | 233.10 |
| SQM 6 | 6/11/13 | 43.723806 | -71.559556 | Mill Brook | 12.15 | 1.64 | 2.48 | 74.70 | 10.27 | 10.91 | 0.00 | 210.40 |
| SRM 6 | 6/11/13 | 43.988306 | -71.327444 | Swift River | 130.87 | 1.06 | 0.00 | 95.95 | 0.10 | 2.84 | 0.05 | 617.30 |
| SRN 6 | 6/11/13 | 43.391472 | -72.186306 | Sugar River | 210.98 | 5.95 | 3.12 | 81.62 | 2.05 | 6.64 | 0.62 | 401.00 |
| SRS 6 | 6/11/13 | 43.389487 | -72.19456 | Sugar River | 325.30 | 7.62 | 5.34 | 74.71 | 7.61 | 4.34 | 0.37 | 399.00 |
| SRU 6 | 6/11/13 | 44.023588 | -71.435663 | Swift River | 12.02 | 3.35 | 0.00 | 96.45 | 0.09 | 0.11 | 0.00 | 781.80 |
| WBG 6 | 6/11/13 | 43.0165 | -71.60125 | Piscataquog (Main) | 5.34 | 8.40 | 2.64 | 84.59 | 0.13 | 4.26 | 0.00 | 193.90 |
| WHB 6 | 6/11/13 | 43.122344 | -71.004961 | Wednesday Hill Brook | 1.27 | 24.47 | 8.96 | 60.30 | 0.00 | 6.28 | 0.00 | 47.90 |
| WIN 6 | 6/11/13 | 43.440067 | -71.651669 | Winnipesaukee | 1261.75 | 8.17 | 2.81 | 64.03 | 19.06 | 5.64 | 0.29 | 220.00 |
| ARD 7 | 7/16/13 | 44.460618 | -71.186188 | Androscoggin | 3503.76 | 1.32 | 0.11 | 85.99 | 6.27 | 5.78 | 0.50 | 576.70 |
| ARU 7 | 7/16/13 | 44.503668 | -71.156587 | Androscoggin | 3437.53 | 1.11 | 0.11 | 86.09 | 6.37 | 5.83 | 0.47 | 578.80 |
| BAT 7 | 7/16/13 | 43.070944 | -70.783111 | Borthwick Ave | 10.59 | 81.69 | 1.01 | 7.61 | 0.14 | 9.55 | 0.00 | 14.90 |
| BBD 7 | 7/16/13 | 42.923317 | -72.2726 | Beaver Brook | 24.14 | 18.10 | 1.89 | 75.18 | 0.09 | 4.64 | 0.09 | 279.70 |
| BBK 7 | 7/16/13 | 42.907736 | -71.922104 | Bogle Brook | 10.72 | 3.43 | 3.79 | 88.55 | 0.39 | 3.85 | 0.00 | 364.80 |
| BBU 7 | 7/16/13 | 42.957194 | -72.266417 | Beaver Brook | 16.99 | 5.85 | 2.17 | 85.84 | 0.06 | 5.95 | 0.13 | 311.00 |
| BBW 7 | 7/16/13 | 42.930722 | -72.271444 | Beaver Brook | 21.89 | 14.67 | 2.06 | 78.09 | 0.05 | 5.04 | 0.10 | 288.00 |
| BDC 5 | 5/14/13 | 43.094303 | -70.987578 | Burley Demerrit | 0.23 | 5.95 | 41.27 | 39.68 | 0.00 | 13.10 | 0.00 | 34.70 |
| BDC 7 | 7/16/13 | 43.094303 | -70.987578 | Burley Demerrit | 0.23 | 5.95 | 41.27 | 39.68 | 0.00 | 13.10 | 0.00 | 34.70 |
| BDD 7 | 7/16/13 | 43.549972 | -72.26775 | Blood Brook | 33.39 | 3.35 | 6.83 | 86.37 | 0.06 | 3.35 | 0.03 | 439.50 |
| BDU 7 | 7/16/13 | 43.546528 | -72.270667 | Blood Brook | 31.49 | 3.35 | 6.67 | 86.46 | 0.07 | 3.42 | 0.03 | 445.30 |
| BLD 7 | 7/16/13 | 45.050556 | -71.385694 | Back Lake Brook | 6.35 | 6.95 | 0.00 | 73.72 | 11.31 | 7.92 | 0.10 | 484.50 |
| BLU 7 | 7/16/13 | 45.052389 | -71.383472 | Back Lake Brook | 6.19 | 6.39 | 0.00 | 74.12 | 11.44 | 7.98 | 0.10 | 485.40 |
| BRD 7 | 7/16/13 | 43.1795 | -70.887 | Bellamy River | 67.60 | 13.11 | 6.90 | 55.02 | 4.05 | 18.19 | 2.72 | 65.10 |
| BRU 7 | 7/16/13 | 43.178861 | -70.890778 | Bellamy River | 66.98 | 25.28 | 15.24 | 38.89 | 0.04 | 14.13 | 6.41 | 65.50 |
| BWS 7 | 7/16/13 | 42.924361 | -72.270389 | Louisville Brook | 7.51 | 0.92 | 0.00 | 99.02 | 0.00 | 0.06 | 0.00 | 515.00 |
| CBT 7 | 7/16/13 | 43.771667 | -71.72989 | Clay Brook | 11.14 | 1.84 | 4.63 | 86.36 | 0.00 | 6.97 | 0.19 | 316.80 |
| CBU 7 | 7/16/13 | 43.7454 | -71.719 | Clay Brook | 7.49 | 1.29 | 5.17 | 89.28 | 0.00 | 4.26 | 0.00 | 359.20 |
| CCK 7 | 7/16/13 | 42.7692 | -70.9165 | Cart Creek | 1.66 | 4.10 | 5.23 | 46.76 | 0.00 | 43.91 | 0.00 | 16.20 |
| CRD 7 | 7/16/13 | 43.800917 | -71.437472 | Creamery Brook | 2.15 | 11.61 | 6.97 | 78.90 | 0.00 | 2.53 | 0.00 | 246.10 |
| CRU 7 | 7/16/13 | 43.803889 | -71.442278 | Creamery Brook | 1.68 | 8.43 | 0.00 | 87.39 | 0.00 | 4.19 | 0.00 | 258.60 |
| CSP 7 | 7/16/13 | 42.5495 | -71.0909 | Cedar Swamp | 3.16 | 24.66 | 1.69 | 24.66 | 0.00 | 43.16 | 0.72 | 28.50 |
| CTC 7 | 7/16/13 | 45.022083 | -71.46275 | Connecticut | 671.17 | 2.07 | 0.36 | 88.51 | 3.82 | 4.76 | 0.36 | 588.40 |
| CTP 7 | 7/16/13 | 45.061611 | -71.298806 | Connecticut | 301.35 | 2.59 | 0.04 | 85.54 | 5.60 | 5.60 | 0.57 | 638.50 |
| DCF 7 | 7/16/13 | 43.13483 | -71.18219 | Dowst-Cate | 7.17 | 5.35 | 3.40 | 77.77 | 1.72 | 11.64 | 0.13 | 152.80 |
| EMB 7 | 7/16/13 | 44.737083 | -71.439722 | Emerson Brook | 1.09 | 0.00 | 0.00 | 100.00 | 0.00 | 0.00 | 0.00 | 829.00 |
| EXT 7 | 7/16/13 | 42.982847 | -70.949131 | Exeter River | 283.19 | 12.22 | 11.40 | 54.79 | 0.81 | 19.84 | 0.93 | 62.00 |
| HBF 7 | 7/16/13 | 43.95305 | -71.72398 | Paradise Brook | 0.67 | 0.00 | 0.00 | 100.00 | 0.00 | 0.00 | 0.00 | 622.10 |
| HOB 7 | 7/16/13 | 43.082111 | -70.796 | Hodgson Brook | 5.23 | 91.18 | 0.00 | 3.30 | 0.00 | 5.50 | 0.00 | 20.60 |
| IDM 7 | 7/16/13 | 42.6597 | -70.8942 | Ipswich Dam | 317.17 | 37.10 | 2.13 | 35.33 | 2.21 | 22.86 | 0.36 | 32.90 |
| IRD 7 | 7/16/13 | 44.411635 | -71.496001 | Israel River | 186.38 | 2.68 | 2.05 | 89.96 | 0.06 | 4.64 | 0.00 | 625.10 |
| IRU 7 | 7/16/13 | 44.361415 | -71.397224 | Israel River | 53.38 | 1.80 | 0.01 | 95.32 | 0.00 | 0.80 | 2.08 | 742.30 |
| JOB 7 | 7/16/13 | 44.758194 | -71.427028 | Johnson Brook | 1.74 | 0.00 | 0.00 | 100.00 | 0.00 | 0.00 | 0.00 | 822.70 |
| LND 7 | 7/16/13 | 44.04173 | -71.664205 | Pemigewasset (East) | 297.85 | 0.84 | 0.01 | 98.39 | 0.07 | 0.54 | 0.15 | 798.80 |
| LNU 7 | 7/16/13 | 44.063814 | -71.590948 | Pemigewasset (East) | 215.59 | 0.08 | 0.00 | 99.12 | 0.05 | 0.56 | 0.18 | 840.30 |
| MMR 7 | 7/16/13 | 43.454417 | -71.227917 | Merrymeeting River | 88.66 | 6.31 | 2.47 | 73.41 | 6.45 | 11.20 | 0.16 | 230.20 |
| MOD 7 | 7/16/13 | 44.373481 | -71.296647 | Moose River | 21.50 | 3.23 | 0.00 | 93.49 | 0.00 | 1.30 | 1.99 | 810.70 |
| MOU 7 | 7/16/13 | 44.373392 | -71.298639 | Moose River | 15.00 | 4.63 | 0.12 | 93.42 | 0.00 | 1.83 | 0.00 | 589.20 |
| MRC 7 | 7/16/13 | 43.940361 | -71.511194 | Mad River | 65.16 | 3.15 | 0.00 | 96.45 | 0.06 | 0.32 | 0.03 | 755.70 |
| MRL 7 | 7/16/13 | 43.969111 | -71.50725 | Mad River | 15.51 | 0.52 | 0.00 | 99.00 | 0.16 | 0.32 | 0.00 | 807.80 |
| NEA 7 | 7/16/13 | 43.176972 | -71.831222 | Contoocook | 974.28 | 6.29 | 3.94 | 77.60 | 3.21 | 8.75 | 0.22 | 342.90 |
| NEB 7 | 7/16/13 | 43.178361 | -71.822028 | Contoocook | 976.38 | 6.33 | 3.94 | 77.57 | 3.21 | 8.74 | 0.22 | 342.60 |
| NED 7 | 7/16/13 | 43.17375 | -71.816833 | Contoocook | 976.28 | 6.37 | 3.94 | 77.54 | 3.21 | 8.74 | 0.21 | 342.60 |
| NEU 7 | 7/16/13 | 43.173472 | -71.817472 | Contoocook | 975.89 | 6.35 | 3.94 | 77.55 | 3.21 | 8.74 | 0.21 | 342.60 |
| NSB 7 | 7/16/13 | 42.740444 | -71.471861 | Salmon Brook | 68.25 | 21.85 | 7.48 | 51.09 | 4.02 | 15.23 | 0.33 | 73.60 |
| NWD 7 | 7/16/13 | 43.081194 | -70.797917 | Newfields Ditch | 3.60 | 95.26 | 0.00 | 0.89 | 0.00 | 3.85 | 0.00 | 22.20 |
| OBG 7 | 7/16/13 | 42.917456 | -71.916053 | Otter Brook | 30.04 | 5.03 | 4.65 | 71.99 | 3.35 | 14.64 | 0.34 | 272.90 |
| OSS 7 | 7/16/13 | 43.79239 | -70.99248 | Ossipee River | 904.48 | 4.26 | 1.47 | 83.24 | 3.59 | 6.73 | 0.70 | 293.40 |
| PBB 7 | 7/16/13 | 43.994875 | -71.352244 | Pine Bend Brook | 2.64 | 0.00 | 0.00 | 87.60 | 0.48 | 11.93 | 0.00 | 469.20 |
| PBD 7 | 7/16/13 | 42.969694 | -71.697861 | Piscataquog | 140.97 | 5.83 | 4.68 | 79.95 | 0.87 | 8.21 | 0.45 | 258.90 |
| PBI 7 | 7/16/13 | 42.978833 | -71.473139 | Piscataquog | 403.63 | 9.11 | 5.65 | 75.70 | 1.26 | 7.76 | 0.52 | 205.20 |
| PBU 7 | 7/16/13 | 42.979889 | -71.689139 | Piscataquog | 143.12 | 6.07 | 4.77 | 79.71 | 0.86 | 8.13 | 0.45 | 257.60 |
| PDM 7 | 7/16/13 | 42.7528 | -70.9461 | Parker Dam | 54.26 | 18.54 | 6.60 | 49.01 | 1.91 | 23.89 | 0.05 | 36.40 |
| PIN 7 | 7/16/13 | 43.76196 | -71.13488 | Pine River | 227.30 | 4.65 | 1.48 | 80.05 | 2.63 | 9.99 | 1.20 | 234.40 |
| PRF 7 | 7/16/13 | 43.437833 | -71.652333 | Pemigewasset | 2646.46 | 3.35 | 1.91 | 89.30 | 2.62 | 2.59 | 0.22 | 447.60 |
| PRG 7 | 7/16/13 | 43.017306 | -71.601167 | Piscataquog (Main) | 332.12 | 6.40 | 5.50 | 78.27 | 1.00 | 8.27 | 0.57 | 218.90 |
| PRP 7 | 7/16/13 | 43.760225 | -71.686503 | Pemigewasset | 1601.99 | 3.12 | 1.25 | 93.06 | 0.42 | 1.88 | 0.27 | 543.70 |
| PRW 7 | 7/16/13 | 44.037967 | -71.686885 | Pemigewasset | 86.66 | 4.49 | 0.02 | 93.25 | 0.22 | 1.36 | 0.66 | 729.80 |
| PWU 7 | 7/16/13 | 43.109028 | -71.762039 | Piscataquog (North) | 79.12 | 5.21 | 5.38 | 77.82 | 4.27 | 7.21 | 0.10 | 284.10 |
| SBK 7 | 7/16/13 | 42.5236 | -71.185 | Sawmill Brook | 4.09 | 87.85 | 0.00 | 8.21 | 0.00 | 3.01 | 0.00 | 47.90 |
| SBM 7 | 7/16/13 | 43.170561 | -71.217811 | Saddleback | 0.49 | 6.32 | 0.00 | 93.68 | 0.00 | 0.00 | 0.00 | 256.50 |
| SCD 7 | 7/16/13 | 44.88275 | -71.071956 | Dead Diamond River | 217.17 | 0.27 | 0.00 | 95.37 | 0.06 | 4.27 | 0.03 | 632.50 |
| SCS 7 | 7/16/13 | 44.881867 | -71.072094 | Swift Diamond River | 178.68 | 0.43 | 0.00 | 95.97 | 0.67 | 2.88 | 0.05 | 664.60 |
| SHB 7 | 7/16/13 | 42.954694 | -71.793389 | School House Brook | 1.50 | 4.70 | 0.48 | 94.52 | 0.00 | 0.30 | 0.00 | 333.20 |
| SLB 7 | 7/16/13 | 44.665389 | -71.457417 | Slide Brook | 1.37 | 0.00 | 0.00 | 98.40 | 0.00 | 0.00 | 1.60 | 665.50 |
| SQB 7 | 7/16/13 | 43.703639 | -71.501333 | Unnamed | 3.13 | 3.03 | 2.98 | 91.33 | 0.20 | 2.46 | 0.00 | 233.10 |
| SQM 7 | 7/16/13 | 43.723806 | -71.559556 | Mill Brook | 12.15 | 1.64 | 2.48 | 74.70 | 10.27 | 10.91 | 0.00 | 210.40 |
| SQR 7 | 7/16/13 | 42.981383 | -70.944344 | Squamscott River | 283.09 | 12.18 | 11.41 | 54.81 | 0.81 | 19.84 | 0.93 | 62.00 |
| SRM 7 | 7/16/13 | 43.988306 | -71.327444 | Swift River | 130.87 | 1.06 | 0.00 | 95.95 | 0.10 | 2.84 | 0.05 | 617.30 |
| SRN 7 | 7/16/13 | 43.391472 | -72.186306 | Sugar River | 210.98 | 5.95 | 3.12 | 81.62 | 2.05 | 6.64 | 0.62 | 401.00 |
| SRS 7 | 7/16/13 | 43.389487 | -72.19456 | Sugar River | 325.30 | 7.62 | 5.34 | 74.71 | 7.61 | 4.34 | 0.37 | 399.00 |
| SRU 7 | 7/16/13 | 44.023588 | -71.435663 | Swift River | 12.02 | 3.35 | 0.00 | 96.45 | 0.09 | 0.11 | 0.00 | 781.80 |
| WBG 7 | 7/16/13 | 43.0165 | -71.60125 | Piscataquog (Main) | 5.34 | 8.40 | 2.64 | 84.59 | 0.13 | 4.26 | 0.00 | 193.90 |
| WHB 7 | 7/16/13 | 43.122344 | -71.004961 | Wednesday Hill Brook | 1.27 | 24.47 | 8.96 | 60.30 | 0.00 | 6.28 | 0.00 | 47.90 |
| WIN 7 | 7/16/13 | 43.440067 | -71.651669 | Winnipesaukee | 1261.75 | 8.17 | 2.81 | 64.03 | 19.06 | 5.64 | 0.29 | 220.00 |

**Table C. Dissolved and total sample site-specific information.** Re-sampled dissolved and total samples analyzed for ions comprising a subset of our sites in northern New England. Samples indicated as ‘bdl’ are due to concentrations below detection limits.

| Site ID | Date | Latitude | Longitude | River | Sodium, mg/L | Iron, mg/L | Copper, mg/L | Zinc, mg/L | Arsenic, mg/L | Cadmium, mg/L | Lead, mg/L |
| --- | --- | --- | --- | --- | --- | --- | --- | --- | --- | --- | --- |
| bdc glass | 8/30/17 | 43.094303 | -70.987578 | Burley Demerrit | 17.2 | 0.257 | 0.00143 | bdl | 0.0000190 | 0.0000202 | bdl |
| bdc plastic | 8/30/17 | 43.094303 | -70.987578 | Burley Demerrit | 15.9 | 0.779 | 0.00342 | 0.00708 | 0.000238 | 0.00000395 | 0.000437 |
| hbf glass | 8/30/17 | 43.95305 | -71.72398 | Hubbard Brook | 2.80 | 0.058 | 0.000287 | bdl | bdl | bdl | bdl |
| hbf plastic | 8/30/17 | 43.95305 | -71.72398 | Hubbard Brook | 2.00 | bdl | 0.000211 | bdl | bdl | 0.00000299 | bdl |
| hob glass | 8/30/17 | 43.08211111 | -70.796 | Hodgson Brook | 139 | 0.241 | 0.00331 | bdl | 0.00372 | 0.00000271 | 0.0000371 |
| hob plastic | 8/30/17 | 43.08211111 | -70.796 | Hodgson Brook | 134 | 0.529 | 0.00458 | bdl | 0.00362 | 0.00000921 | 0.000149 |
| nwd glass | 8/30/17 | 43.081194 | -70.797917 | Newfields Ditch | 114 | 0.219 | 0.00296 | bdl | 0.00172 | 0.00000983 | 0.000125 |
| nwd plastic | 8/30/17 | 43.081194 | -70.797917 | Newfields Ditch | 123 | 2.95 | 0.00635 | 0.0111 | 0.00558 | 0.0000359 | 0.000411 |
| pbb glass | 8/30/17 | 43.994875 | -71.352244 | Pine Bend Brook | 2.65 | 0.0350 | bdl | bdl | bdl | bdl | bdl |
| pbb plastic | 8/30/17 | 43.994875 | -71.352244 | Pine Bend Brook | 1.69 | bdl | bdl | bdl | bdl | 0.00000740 | bdl |
| prp glass | 8/30/17 | 43.760225 | -71.686503 | Pemigewasset | 14.9 | 0.103 | 0.00205 | bdl | bdl | bdl | bdl |
| prp plastic | 8/30/17 | 43.760225 | -71.686503 | Pemigewasset | 14.4 | 0.279 | 0.000441 | bdl | bdl | 0.0000116 | 0.0000386 |
